# LncRNA CASC9 interacts with CPSF3 to regulate TGF-β signaling in colorectal cancer

**DOI:** 10.1186/s13046-019-1263-3

**Published:** 2019-06-11

**Authors:** Kaili Luo, Jingwen Geng, Qinkai Zhang, Yesha Xu, Xunzhu Zhou, Zheng Huang, Ke-Qing Shi, Chenwei Pan, Jianmin Wu

**Affiliations:** 1 Institute of Genomic Medicine, Wenzhou Medical University, 268 Xueyuan Road, Wenzhou, 325000 Zhejiang, People’s Republic of China; 2 Key Laboratory of Diagnosis and Treatment of Severe Hepato-Pancreatic Diseases of ZheJiang Province, Wenzhou, 325000 Zhejiang, People’s Republic of China; 3 Center of Precision medicine, The First Affiliated Hospital of Wenzhou Medical University, Wenzhou, 325000 Zhejiang, People’s Republic of China; 4 Department of Infectious Disease, The Second Affiliated Hospital and Yuying Children of Wenzhou Medical University, Wenzhou, 325027 Zhejiang, People’s Republic of China; 5 Pediatric Hepatitis & Liver disease Clinical Center, The Second Affiliated Hospital and Yuying Children of Wenzhou Medical University, Wenzhou, 325027 Zhejiang, People’s Republic of China

**Keywords:** CASC9, CPSF3, TGFβ2, Colorectal cancer

## Abstract

**Background:**

Colorectal cancer (CRC) is the third most frequent cancer and the second leading cause of cancer-related death worldwide. Increasing evidence indicates that the deregulation of long noncoding RNAs (lncRNAs) contributes to tumor initiation and progression; however, little is known about the biological role of cancer susceptibility candidate 9 (CASC9) in CRC.

**Methods:**

Novel lncRNAs potentially involved in CRC tumorigenesis were identified from datasets downloaded from The Cancer LncRNome Atlas and The Atlas of Noncoding RNAs in Cancer. The CRC cell lines HCT-116, HCT-116 p53^−/−^, SW620, SW480, HT-29, LoVo, LS-174T, and RKO were used. Colony-formation, MTS, cell-cycle, apoptosis, and in-vivo tumorigenesis assays were used to determine the role of CASC9 in CRC cell growth in vitro and in vivo. Potential interaction between CASC9 and cleavage and polyadenylation specificity factor subunit 3 (CPSF3) was evaluated using RNA immunoprecipitation and RNA-protein pull-down assays. RNA-sequencing was performed to analyze gene expression following CASC9 knockdown. RT-qPCR, western blotting, and mRNA decay assays were performed to study the mechanisms involved.

**Results:**

CASC9 was frequently upregulated in CRC, which was correlated with advanced TNM stage, and higher CASC9 levels were associated with poor patient outcomes. Knockdown of CASC9 inhibited growth and promoted apoptosis in CRC cells, whereas ectopic CASC9 expression promoted cell growth in vitro and in vivo. We demonstrated that CPSF3 is a CASC9-interacting protein, and knockdown of CPSF3 mimicked the effects of CASC9 knockdown in CRC cells. Furthermore, we found that CASC9 exerts its oncogenic activity by modulating TGFβ2 mRNA stability and upregulating the levels of TGFβ2 and TERT, resulting in an increase in phosphorylated SMAD3 and activation of TGF-β signaling, and enhanced TERT complex function in CRC cells. Finally, CPSF3 was significantly upregulated in CRC tissues as compared with adjacent or non-adjacent normal colon tissues, and CASC9, CPSF3, and TGFβ2 levels in human CRC tissues were positively correlated.

**Conclusions:**

CASC9 is a promising prognostic predictor for patients with CRC and the CASC9-CPSF3-TGFβ2 axis is a potential therapeutic target for CRC treatment.

**Electronic supplementary material:**

The online version of this article (10.1186/s13046-019-1263-3) contains supplementary material, which is available to authorized users.

## Background

Colorectal cancer (CRC) is the third most frequent cancer and the second leading cause of cancer-related death worldwide [1–3]. As in many other solid tumors, sequential genetic and epigenetic changes in specific oncogenes and/or tumor suppressor genes can induce CRC onset, progression, and metastasis [4]. Very early diagnosis and personalized care, as well as a better knowledge of the molecular basis of disease onset and progression are crucial to developing a cure for CRC. Thus, a better understanding of the mechanisms that drive the disease and the identification of novel therapeutic targets are priorities for improving CRC treatment.

The characterization of specific changes in the cancer transcriptome may facilitate the development of new therapeutic strategies in CRC. A genome-wide transcriptional profiling analysis revealed that more than 68% (58,648) of genes are classified as long non-coding RNAs (lncRNAs), 79% of which were previously unannotated [5]. Genome-wide association studies of tumor samples have identified numerous lncRNAs associated with various types of cancer, including CRC [6]. The majority of lncRNAs are expressed in a highly tissue- and cell type-specific manner [7, 8] and therefore, are potentially efficacious targets for systemic cancer treatment [9].

LncRNAs are commonly categorized based on their location and function [10, 11]. Alterations in lncRNAs expression and their mutations promote tumorigenesis and metastasis [12–14]. In CRC, several lncRNAs have been implicated in chromatin remodeling and transcriptional and posttranscriptional gene expression regulation [6]. The human chromosome region 8q24 reportedly encodes at least four lncRNAs expressed in CRC samples. For example, colon cancer-associated transcript-1 (CCAT1) is upregulated by MYC in CRC and is associated with regional and distant liver metastasis [15]. CCAT1-L is located in the 8q24.21 region, upstream of MYC, and acts as an enhancer in CRC cells. It assists in maintaining chromatin looping by modulating the binding of CTCF to the MYC locus [16]. CCAT2 is another lncRNA located within 8q24; it encompasses the single nucleotide polymorphism rs6983267, which is involved in chromosomal instability, resulting in increased proliferation and metastasis in microsatellite stable tumors [17, 18]. PCAT-1, also located in 8q24, is overexpressed in CRC and is associated with distant metastasis and overall survival [19]. Recent data from The Cancer LncRNome Atlas (TCLA) indicate that more than 800 lncRNAs are dysregulated in CRC (http://tcla.fcgportal.org/) [20]. However, few of them have been studied in CRC. Thus, there remain gaps in our knowledge about the functions of lncRNAs in CRC.

In the present study, we aimed to identify and characterize lncRNAs that are functionally important in CRC. We conducted a combined analysis of lncRNA expression data in CRC and normal tissues as well as clinical survival data downloaded from TCLA and The Atlas of Noncoding RNAs in Cancer (TANRIC) [5], respectively. In-vitro and in-vivo analyses were carried out to identify the roles and mechanisms of candidate lncRNAs in CRC.

## Methods

### Cell culture and clinical specimens

Human CRC cell lines HCT-116, HCT-116 p53^−/−^, SW620, SW480, HT-29, LoVo, LS-174T, and RKO, and HEK293T cells were obtained from the American Type Culture Collection (ATCC, Manassas, VA, USA). HCT-116, HCT-116 p53^−/−^, SW620, SW480, and HT-29 cells were maintained in McCoy’s 5A medium (Gibco, Grand Island, NY, USA). HEK293T, LS-174T, and RKO cells were cultured in Dulbecco’s modified Eagle’s medium (Biological Industries, Beit HaEmek, Israel). LoVo cells were cultured in RPMI 1640 medium (Biological Industries). Media were supplemented with 10% fetal bovine serum (Gibco) and 1% penicillin–streptomycin mixture and the cells were cultured at 37 °C in a 5% CO_2_ incubator. All cell lines were free of mycoplasma and were authenticated by genetic profiling using polymorphic short tandem repeat loci. Forty paired fresh primary colorectal tumors and matched adjacent normal tissues were obtained with patient informed consent and approval from the Institutional Ethics Committee of the Second Affiliated Hospital of Wenzhou Medical University.

### Small interfering RNAs (siRNAs), GapmeRs, and transfection

SiRNAs were synthesized by GenePharma (Shanghai, China). Antisense LNA™ GapmeRs targeting all four transcript variants of CASC9 and a negative control were obtained from Qiagen (Valencia, CA, USA). SiRNAs and GapmeRs were transfected at a final concentration of 20 nM and 10 nM, respectively, using Lipofectamine 2000 (Life Technologies, Carlsbad, CA, USA) according to the manufacturer’s instructions. Cells were harvested 48 h after transfection for subsequent analysis. The siRNA and GapmeR sequences are listed in Additional file 1: Table S1.

### Plasmids

To construct CASC9 overexpression plasmids (LV-CASC9–202 and LV-CASC9–204), PCR products were inserted into the *Xho*I/*Xba*I sites of the pLVX-IRES-puro vector (Addgene, Watertown, MA, USA) using an In-Fusion HD Cloning Plus kit (TaKaRa, Dalian, China) per the manufacturer’s instructions. To construct CASC9 knockdown plasmids (shCASC9–1 and shCASC9–2), annealed oligos containing the target sequences were inserted into the *Age*I/*EcoR*I sites of the pLKO.1-TRC cloning vector (Addgene). The vectors psPAX2 and pMD2.G were obtained from Addgene. The pcDNA3.1-CAC9–202 and pcDNA3.1-CAC9–204 vector was synthesized by TSINGKE (Hangzhou, China). The oligos used for cloning were obtained from TSINGKE and are presented in Additional file 1: Table S1.

### Generation of stable cell lines

To generate stable CASC9-knockdown or CASC9-overexpressing cell lines, lentiviral particles were produced in HEK293T cells cotransfected with shCASC9–1, shCASC9–2, LV-CASC9–202, LV-CASC9–204, or the corresponding empty vectors, and psPAX2 and pMD2.G plasmids. Then, HCT-116, SW620, and SW480 cells were infected with lentiviral particles in the presence of 8 μg/ml polybrene (Genechem, Shanghai, China) and selected using 0.5 μg/ml puromycin. After 1 week, puromycin-resistant cell pools were collected and verified by reverse-transcription quantitative PCR (RT-qPCR).

### Cytoplasmic and nuclear RNA isolation

Cytoplasmic and nuclear RNA was extracted using the Nuclear and Cytoplasmic Protein Extraction Kit (Beyotime, Shanghai, China) and TRIzol reagent (Thermo Fisher Scientific, Waltham, MA, USA) according to the manufacturer’s instructions. CASC9 expression was measured by RT-qPCR.

### RT-qPCR

Total RNA was extracted using TRIzol reagent and was treated with RQ1 RNase-Free DNase (Promega, Madison, WI, USA) for 30 min. cDNA was synthesized using the M-MLV reverse transcription kit (Promega) according to the manufacturer’s instructions. qPCR was conducted using ChamQ Universal SYBR qPCR Master Mix (Vazyme Biotech, Nanjing, China) on a QuantStudio 3 instrument (Thermo Fisher Scientific). The mRNA level of the β-actin housekeeping gene served as a control. qPCR primer sequences are listed in Additional file 1: Table S1.

### In-vitro cell proliferation and viability assays

For colony-formation assays, treated cells were seeded into a 6-well plate at 500 cells/well and cultured for 10–14 days. Colonies were fixed with pre-cooled methanol and stained with 0.1% crystal violet, and colonies consisting of more than 50 cells were counted. Cell viability was evaluated by (3-(4,5-dimethylthiazol-2-yl)-5-(3-carboxymethoxyphenyl)-2-(4-sulfophenyl)-2H-tetrazolium (MTS) assays as described previously [21], using the CellTiter 96 AQueous One Solution Cell Proliferation Assay Kit (Promega) according to the manufacturer’s instructions.

### Cell-cycle and apoptosis analyses

Treated cells were harvested at 80% confluence and washed twice with ice-cold phosphate-buffered saline (PBS). For cell-cycle analysis, treated cells were harvested and fixed with pre-cooled 70% ethanol at 4 °C overnight, then washed with ice-cold PBS twice and filtered through a 0.05-mm cell strainer. After incubation with PBS containing 50 μg/mL propidium iodide (PI), 100 μg/mL RNase A, and 0.2% Triton X-100 at 4 °C for 30 min, cells were analyzed for DNA content by flow cytometry on a C6 Plus instrument (BD Biosciences, Franklin Lakes, NJ, USA). For apoptosis analysis, treated cells were harvested with trypsin without EDTA, stained with an FITC/Annexin V Apoptosis Detection Kit (BD Biosciences) at room temperature for 15 min following the manufacturer’s instructions, and subjected to flow cytometry.

### In-vivo tumorigenesis assays

For xenograft studies, 6-week-old male BALB/c nude mice were purchased from Shanghai Laboratory Animal Center (Shanghai, China) and housed under pathogen-free conditions. Logarithmically growing cells were harvested and resuspended in PBS. In total, 1 × 10^7^ (for CASC9-overexpressing SW480 cells, or CASC9-knockdown SW620 cells), 1 × 10^6^ (for CASC9-overexpressing HCT-116 cells), or 2 × 10^6^ (for CASC9-knockdown HCT-116) cells were subcutaneously injected into the rear flanks of the mice. Tumors were measured using a Vernier caliper, and tumor volume was calculated using the following equation: V = L × W^2^ × 0.5236 (L = long axis, W = short axis). After 18–24 days, the mice were sacrificed, and the tumors were harvested and weighed. All animal studies were reviewed and approved by the Institutional Ethics Committee of Wenzhou Medical University.

### Immunohistochemistry

Paraffin sections (5 μm) from tumor tissue samples were deparaffinized in 100% xylene and rehydrated with a decreasing ethanol series and then water. Antigen retrieval was conducted in 0.01 mol/L sodium citrate buffer (pH 6.0) at 100 °C for 10 min. Immunohistochemistry assay was performed as described previously [22] using super-sensitive horseradish peroxidase immunohistochemistry kit (Sangon Biotech, Shanghai, China) per the manufacturer’s instructions, and the anti-Ki67 antibody (sc-23900, Santa Cruz) was used.

### RNA-sequencing

Total RNA was extracted using TRIzol, and RNA quantity and purity were analyzed using a Bioanalyzer 2100 and RNA 1000 Nano LabChip Kit (Agilent Technologies, Santa Clara, CA, USA), with a threshold RNA integrity number of > 7.0. Poly(A) RNA was purified from total RNA (5 μg) using poly-T oligo-attached magnetic beads and two rounds of purification. Following purification, mRNA was fragmented using divalent cations at an elevated temperature. Then, the cleaved RNA fragments were reverse-transcribed into cDNA library in accordance with the protocol for the mRNA-Seq sample preparation kit (Illumina, San Diego, CA, USA). The average insert size for the paired-end libraries was 300 bp (± 50 bp). Paired-end sequencing was conducted on an Illumina HiSeq X10 platform by LC-BIO Technologies CO., LTD. (Hangzhou, China) following the vendor’s recommended protocol. After removing low-quality reads, the clean paired-end reads were submitted to the Gene Expression Omnibus under accession number GSE125648. The HISAT [23] package was used for mapping reads to the UCSC (http://genome.ucsc.edu/) *Homo sapiens* reference genome. StringTie [24] was used to analyze gene expression levels by calculating fragments per kilobase of exon model per million mapped reads. Differentially expressed genes were selected based on a fold change ≥2 or ≤ 0.5 and *P* < 0.05 using the EdgeR package (Bioconductor, http://bioconductor.org/). Gene Ontology (GO) and signaling pathway (based on Biocarta) analyses were conducted in EnrichR [25, 26]. Gene set enrichment analysis (GSEA) was carried out using software from the Broad Institute [27, 28].

### RNA immunoprecipitation (RIP) assay

Cells were washed twice with PBS and harvested by scratching in cold PBS. Then, the cells were resuspended in cell lysis buffer (CLB: 25 mM Tris-HCl at pH 7.4, 150 mM KCl, 1 mM EDTA, 0.5% NP-40, 0.5 mM dithiothreitol, 12 mM β-glycerophosphate, 10 mM NaF, and 2 mM sodium orthovanadate) containing 100 U/mL RNasin ribonuclease inhibitor and protease inhibitor cocktail (Thermo Fisher Scientific). Cell lysates were incubated on ice for 30 min and mixed every 10 min, then centrifuged at 14,000×*g* at 4 °C for 5 min. The supernatants were collected and pre-cleared with Protein A agarose beads (Millipore, Bedford, MA) at 4 °C for 90 min. In parallel, 80 μL Protein A agarose beads were incubated with 4 μg antibody or isotype control at 4 °C for 90 min on a turning wheel. Antibody/bead complex was collected by centrifugation at 1000×*g* at 4 °C for 5 min, and the pre-cleared supernatants were added and incubated overnight at 4 °C on a turning wheel. Next, the antibody/bead complexes were washed three times with washing buffer (50 mM Tris-HCl at pH 7.4, 150 mM KCl, 1 mM EDTA, 0.5% NP-40, 12 mM β-glycerophosphate, 10 mM NaF, 2 mM sodium orthovanadate, 25 U/ml RNasin ribonuclease inhibitor, and protease inhibitor cocktail) and once with PBS at 4 °C on a turning wheel for 5 min each time. Then, the samples were resuspended in 100 μL of CLB and divided into 20 μL for protein analysis and 80 μL for RNA extraction. Glycogen (10 μg) was added to the aqueous phase as a carrier before adding isopropanol to precipitate the RNA.

### RNA pull-down assay

CASC9–202, CASC9–204, antisense-CASC9–202, and antisense-CASC9–204 sequences were amplified by PCR using paired primers containing the T7 promoter sequence at their 5′ end. PCR primer sequences are listed in Additional file 1: Table S1. The PCR products were purified and transcribed using the TranscriptAid T7 High Yield Transcription Kit (Thermo Fisher Scientific) according to the manufacturer’s instructions. The in vitro-transcribed RNA was treated with DNase I, purified, and labeled using the Pierce™ RNA 3′ End Desthiobiotinylation Kit (Thermo Fisher Scientific). HCT-116 cells were harvested and lysed in CLB containing protease inhibitor and RNase inhibitor. RNA pull-down was conducted by binding of the desthiobiotinylated RNA to streptavidin-linked magnetic beads using the Pierce™ Magnetic RNA-Protein Pull-Down Kit (Thermo Fisher Scientific). The RNA-bound protein complex was eluted and analyzed by western blotting.

### Western blotting

Cells were lysed in RIPA buffer, and total protein was quantified using the BCA Protein Assay Kit (Beyotime). Total denatured protein (30 μg) was subjected to sodium dodecyl sulfate-polyacrylamide gels and transferred to polyvinylidene fluoride membranes (Millipore). The blots were incubated with primary antibody at 4 °C overnight, then with horseradish peroxidase-linked secondary antibody at room temperature for 1 h. Immunocomplexes were detected with SuperSignal West Pico Chemiluminescent Substrate (Thermo Fisher Scientific). The primary antibodies anti-polyadenylation specificity factor subunit 3 (CPSF3) (11609–1-AP), anti-EIF4A3 (17504–1-AP), and anti-TGF-β2 (19999–1-AP) were obtained from ProteinTech (Chicago, IL, USA). Anti-SMAD2/3 (#8685), anti-phospho-SMAD3 (#9520), and anti-GAPDH (#2118) were obtained from Cell Signaling Technology (Danvers, MA, USA).

### mRNA decay assay

HCT-116 cells were transfected with siRNA, GapmeR, or the corresponding control. After 48 h, the cells were treated with 5 μg/mL actinomycin D for the indicated time points. Total RNA was extracted, treated with DNase, reverse-transcribed, and quantified by RT-qPCR. After normalizing mRNA levels to that of β-actin, decay rates were calculated by setting the RNA level at 0 h as 100% for both the treated and negative control groups. Exponential fitting curves were determined by the logarithmic least squares method.

### Statistical analysis

Data are presented as the mean ± s.d. and were analyzed in GraphPad Prism 7.0 (GraphPad Software, La Jolla, CA, USA). Unless otherwise noted, each experiment was carried out at least in triplicate. Statistical significance was analyzed by two-tailed Student’s *t*-test, Mann-Whitney *U*-test, or Wilcoxon signed-rank test. Differences between groups were determined using two-way ANOVA. The significance of the clinicopathologic parameters of CRC patients was determined by the chi-squared test. Differences with *P <* 0.05 were considered statistically significant.

## Results

### LncRNA CASC9 is frequently upregulated in CRC

**Fig. 1:**
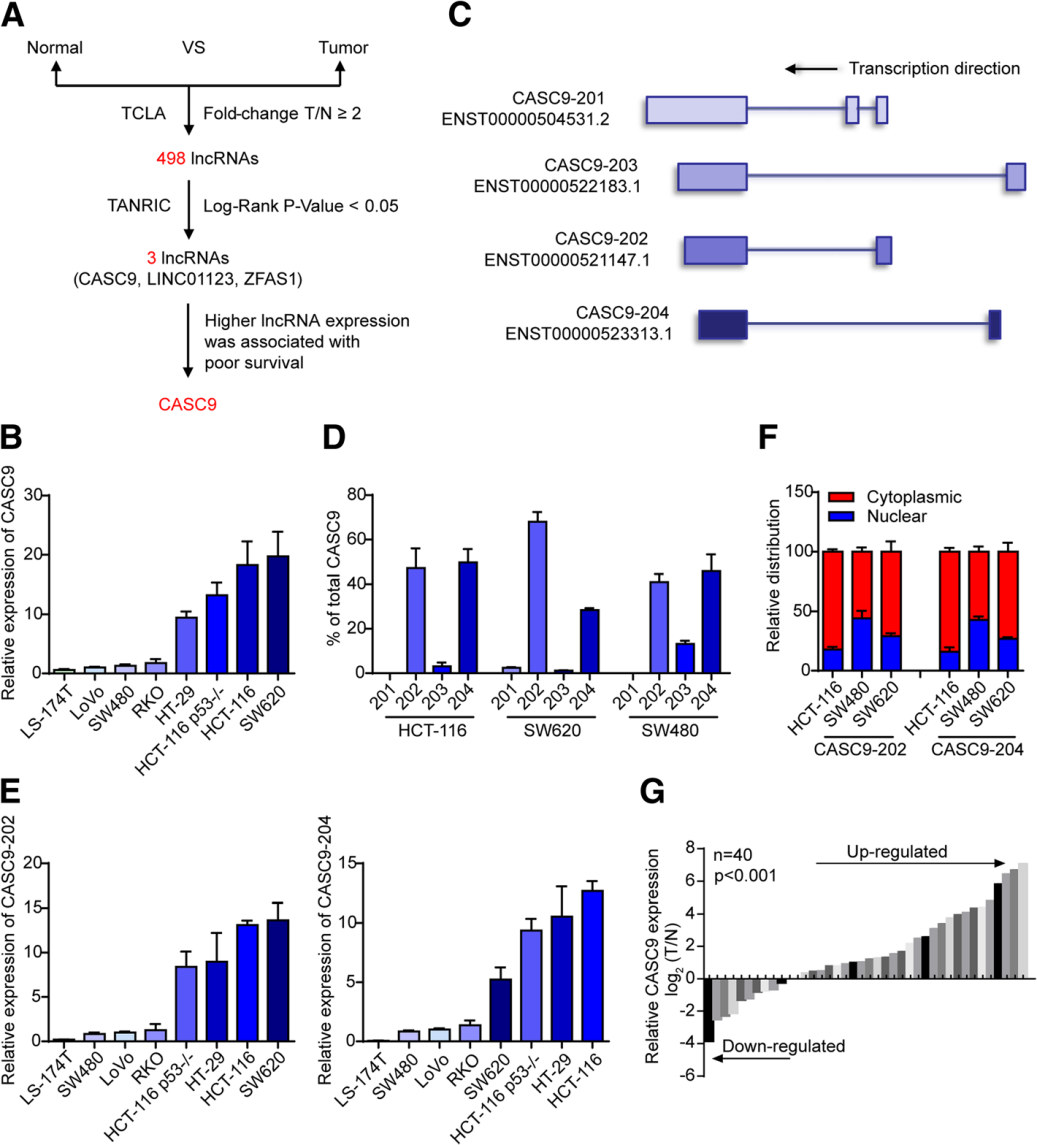
Identification of CASC9 as an lncRNA upregulated in CRC. **a** Schematic representation of the process used to identify upregulated lncRNAs with significant prognostic values in COAD. **b** Expression of CASC9 in CRC cell lines was quantified by RT-qPCR. β-Actin mRNA was used as an internal control. **c** Exon maps for four transcript variants of CASC9 with Ensembl IDs. **d** RNA expression analysis of the four transcript variants of CASC9 in HCT-116, SW620, and SW480 cells by RT-qPCR. **e** RNA expression analysis of the two most abundant CASC9 transcript variants, CASC9–202 (left) and CASC9–204 (right), in CRC cell lines. **f** Subcellular localization of CASC9–202/204 was analyzed by RT-qPCR upon biochemical fractionation of HCT-116, SW480, and SW620 cells. GAPDH mRNA was used as a control for cytoplasmic transcripts, NEAT1 was used as a control for nuclear transcripts. **g** RT-qPCR analysis of relative CASC9 expression in 40 primary CRC tissues and paired normal tissues. The expression of CASC9 was normalized to that of β-actin. The data are presented as the mean ± s.d.

To identify novel lncRNAs involved in CRC tumorigenesis, we downloaded lncRNA expression data for colon adenocarcinoma (COAD) (860 dysregulated lncRNAs, including 498 upregulated and 362 downregulated lncRNAsin COAD; fold change ≥2 or ≤ 0.5, *P* < 0.05) and clinical survival data (104 lncRNAs with significant prognostic value, log-rank *P* < 0.05) from TCLA (http://tcla.fcgportal.org/) and TANRIC [29], respectively. By combined analysis, we identified three upregulated and five downregulated lncRNAs that showed significant prognostic value (Fig. 1a, Additional file 2: Figure S1a). Among them, only CASC9 was upregulated in CRC and was associated with poor patient outcomes (Additional file 2: Figure S1b, c). In normal tissues, CASC9 levels were the highest in the colon and bladder (Additional file 3: Figure S2). Therefore, this lncRNA was selected for further in-depth study.

An initial RT-qPCR survey of eight CRC cell lines revealed that CASC9 expression was substantially higher in SW620, HCT-116, HCT-116 p53^−/−^, and HT-29 cells than in the other cell lines (Fig. 1b). According to Ensembl [30], the CASC9 gene has four transcript variants (Fig. 1c); thus, the relative expression levels of these transcripts were assessed with specific primer pairs. As shown in Fig. 1d, CASC9–202 and CASC9–204 were the most abundant transcript variants in the three cell lines tested (HCT-116, SW620, and SW480). Similar to the expression of total CASC9, high levels of CASC9–202 and CASC9–204 were detected in SW620, HCT-116, HCT-116 p53^−/−^, and HT-29 cells (Fig. 1e). Subcellular localization analysis by biochemical fractionation showed that the two most abundant variants, CASC9–202 and CASC9–204, were present in both the nucleus and cytoplasm, though relative levels varied among cell lines (Fig. 1f), suggesting that they play multiple roles in different cell compartments. Moreover, in 28 out of 40 (70%) primary CRC tumors, CASC9 was significantly upregulated in tumor samples as compared to matched adjacent normal tissues (Fig. 1g). Further statistical analysis revealed a significant association between increased CASC9 expression and advanced TNM stage (Table 1).

**Table 1:** Relationship between CASC9 expression and clinicopathologic parameters of CRC patients

Variables	Patient number (*n* = 40)	CASC9 levels		*P* value	Chi-square
		Low (*n* = 12)	High (*n* = 28)		
Age(years)					
≥ 65	21	4	17	0.112	2.525
< 65	19	8	11		
Gender					
Female	17	5	12	0.944	0.005
Male	23	7	16		
Tumor location					
Colon	30	10	20	0.426	0.635
Rectum	10	2	8		
Tumor size					
≥ 5 cm	7	1	6	0.318	0.998
< 5 cm	33	11	22		
TNM stage					
I	9	6	3	0.020	7.845
II	9	1	8		
III	22	5	17		
Lymph node metastasis					
Presence	17	4	13	0.443	0.590
Absence	23	8	15		
Differentiation grade					
Well	5	2	3	0.159	3.673
Moderate	28	10	18		
Poor	7	0	7		

### Knockdown of CASC9 inhibits growth and promotes apoptosis in CRC cells in vitro and in vivo

To explore the biological role of CASC9 in CRC further, siRNAs targeting CASC9 were utilized to suppress its expression in SW620 cells (Additional file 4: Figure S3a). As expected, knockdown of CASC9 resulted in a significant decrease in cell proliferation and viability as compared with that in control cells as indicated by colony formation and MTS assays (Additional file 4: Figure S3b, c). Furthermore, cell-cycle analysis revealed that knockdown of CASC9 led to cell-cycle arrest in G2/M phase and reduced the cell population in S phase (Additional file 4: Figure S3d).

**Fig. 2:**
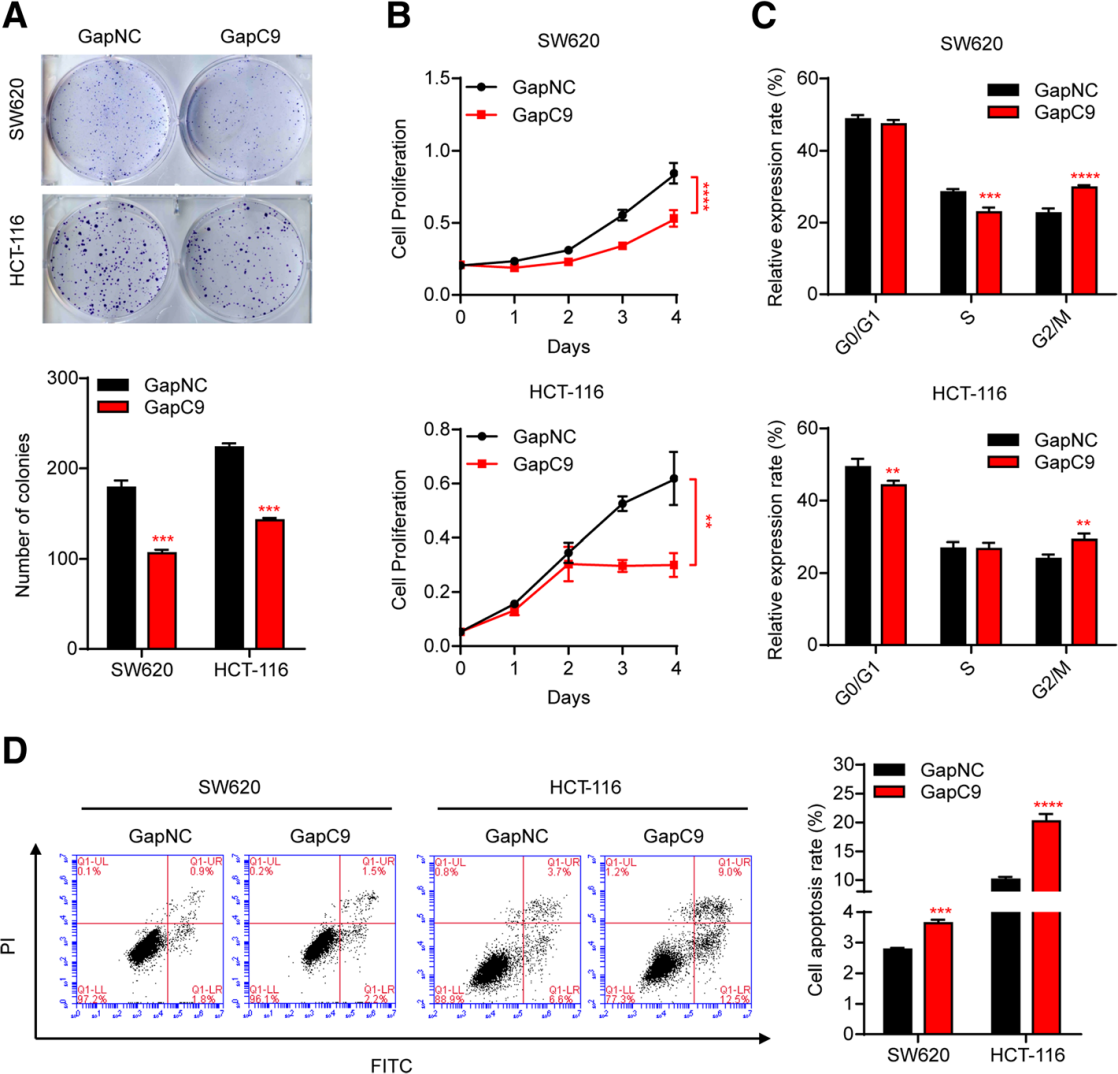
Silencing of CASC9 by GapmeR inhibits CRC cell growth and promotes cell apoptosis in vitro*.* HCT-116 and SW620 cells were transfected with Antisense LNA GapmeR targeting CASC9 (GapC9) or control GapmeR (GapNC). **a, b** Cell proliferation was determined by colony-formation assay **(a)** and, at the indicated time points, by MTS assay **(b)**. **c** Cell cycle was analyzed by flow cytometry. The percentages of cells in G0/G1, S, and G2/M phases are shown. **d** Cell apoptosis was increased in CASC9-knockdown cells as determined by flow cytometry of cells with Annexin V-FITC/PI double staining. The data are presented as the mean ± s.d. **P < 0.01, ***P < 0.001, ****P < 0.0001 by Student’s *t*-test **(a, c, d)** or two-way ANOVA **(b)**

Given that CASC9 was ubiquitously expressed in both the nucleus and cytoplasm of CRC cells and that cytoplasmic lncRNAs are more effectively suppressed by siRNAs, we also silenced CASC9 using Antisense LNA GapmeRs, which have been shown to be highly effective for knockdown of mRNA and lncRNA in an RNase H-dependent manner [31]. As expected, transfection with Antisense LNA GapmeR targeting CASC9 (GapC9) more effectively reduced the levels of CASC9 in HCT116 and SW620 cells (Additional file 5: Figure S4a). More importantly, compared with the cytoplasm-specific reduction in CASC9 by siRNAs, GapC9 transfection suppressed CASC9 expression in both the nucleus and the cytoplasm (Additional file 5: Figure S4b). Subsequently, CRC cell proliferation was assessed in vitro. Silencing of CASC9 by GapmeR blocked CRC cell proliferation as assessed by colony formation assay and decreased cell viability according to MTS assay in both SW620 and HCT-116 cells (Fig. 2a, b). Furthermore, knockdown of CASC9 led to cell-cycle arrest in the G2/M phase (Fig. 2c, Additional file 5: Figure S4c) and promoted apoptosis of SW620 and HCT-116 cells (Fig. 2d).

**Fig. 3:**
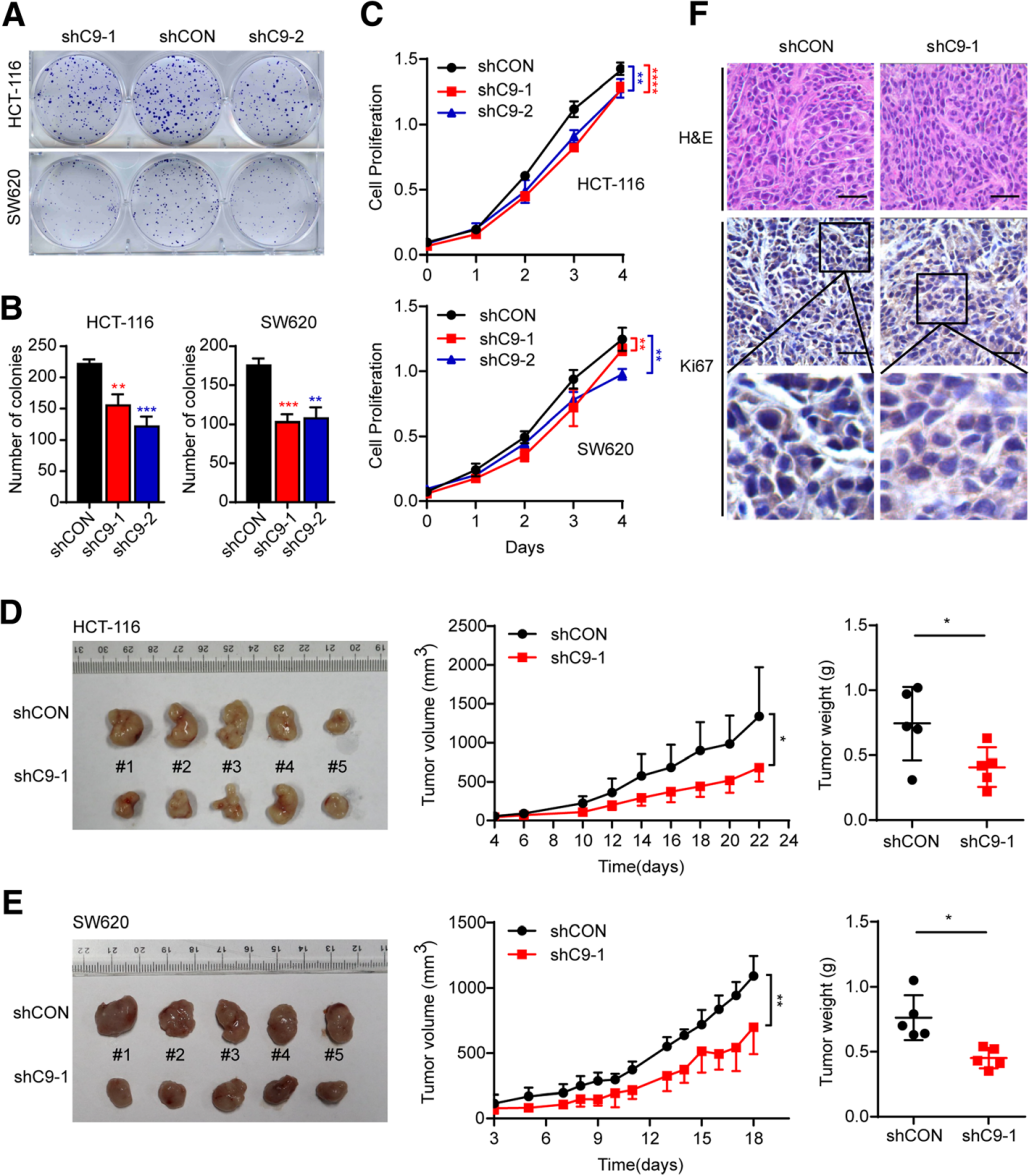
Stable knockdown of CASC9 suppresses CRC cell growth in vivo*.* **a**, **b** Cell growth was determined by colony-formation assay in HCT-116 and SW620 cells transduced with shCASC9 lentivirus (shC9–1 or shC9–2) or control lentivirus (shCON). **a** Representative graphs for colony-formation assay. **b** Quantification of colony numbers. **c** Cell proliferation of shCASC9-transduced HCT-116 and SW620 cells was determined at the indicated time points by MTS assay. **d, e** Subcutaneous xenografts of HCT-116 or SW620 cells infected with shC9–1 lentivirus or shCON lentivirus (*n* = 5). (**d** for HCT-116**, e** for SW620) Images of tumors from nude mice at autopsy are presented (left), the tumor volumes were measured at the indicated time points (middle), and the average weight of the xenografted tumors was measured (right). **f** Immunohistochemical staining of Ki67 in xenografted tumors of mice injected with shCASC9-HCT-116 or control cells. Scale bar, 50 μm. The data are presented as the mean ± s.d. *P < 0.05, **P < 0.01, ***P < 0.001, ****P < 0.0001 by Student’s *t*-test **(b, d, e)** or two-way ANOVA **(c, d, e)**

Next, stable SW620 and HCT-116 CASC9-knockdown cell lines were generated by infecting the cells with two CASC9 shRNA lentiviral vectors. The shRNA targeting efficiency was confirmed by RT-qPCR (Additional file 6: Figure S5a). Accordingly, cell proliferation and viability were decreased in CASC9 shRNA-transduced CRC cells as compared to control cells (Fig. 3a, b, c). To determine whether knockdown of CASC9 affects tumor formation in vivo, we performed an in-vivo tumorigenesis study by subcutaneously injecting shCASC9-HCT-116 or -SW620 cells into nude mice. As expected, the average tumor volumes and weights were significantly lower in mice injected with shCASC9 CRC cells than in those injected with control cells (Fig. 3d, e). Ki-67 staining revealed that tumors from shCASC9 cells had fewer proliferative cells than those in the control group (Fig. 3f). Collectively, these results indicated that silencing of CASC9 significantly inhibits CRC tumorigenesis in vitro and in vivo.

### Ectopic CASC9 expression promotes cell growth in vitro and in vivo

**Fig. 4:**
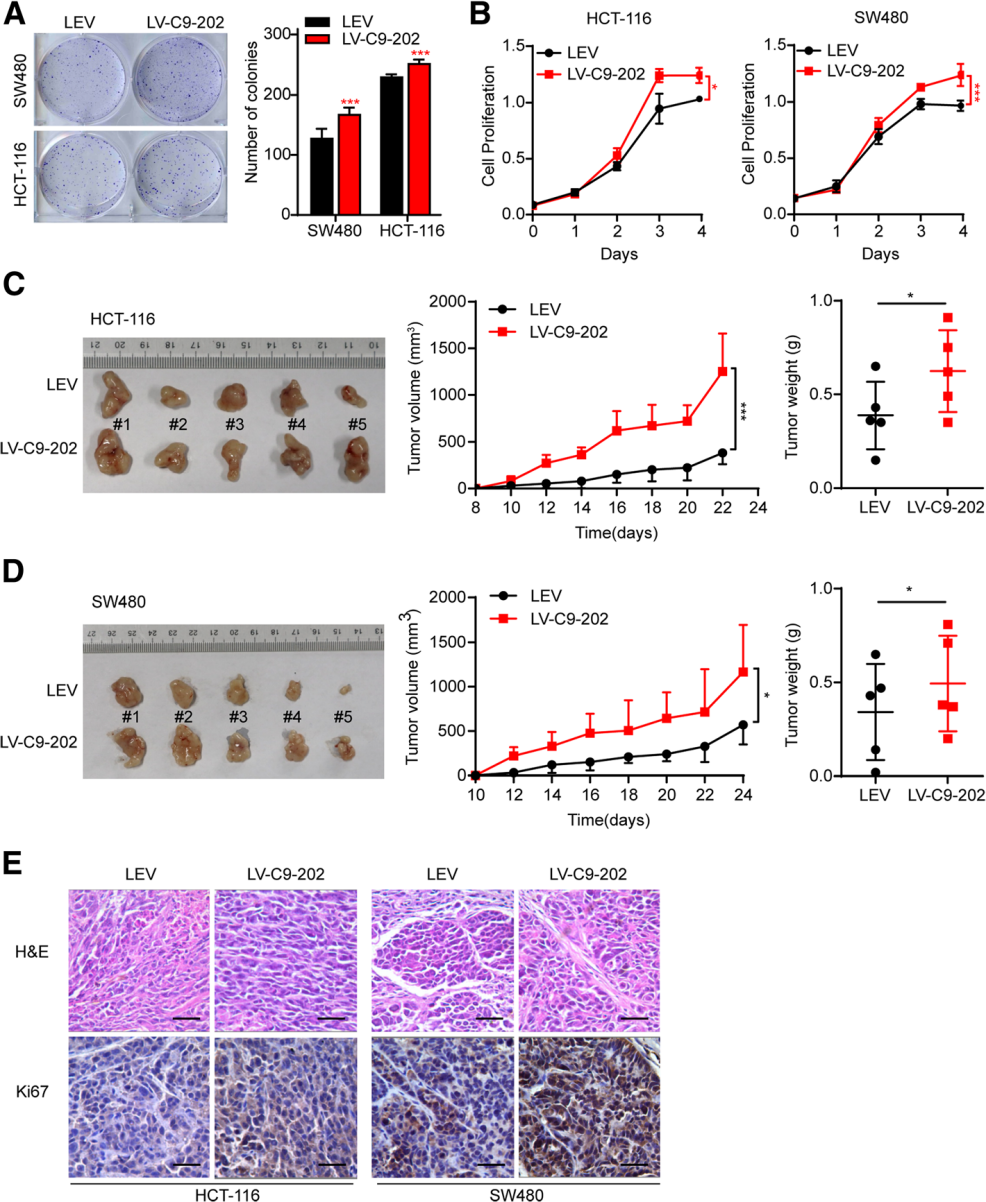
Ectopic CASC9–202 expression promotes CRC cell growth in vitro and in vivo*.* HCT-116 and SW480 cells were transduced with CASC9–202-overexpressing lentivirus (LV-C9–202) or control lentivirus (LEV). **a, b** Cell proliferation was determined by colony-formation assay **(a)** and, at the indicated time points, by MTS assay **(b). c, d** Subcutaneous xenografts of HCT-116 or SW480 cells transduced with LV-C9–202 or LEV (*n* = 5). **(c** for HCT-116**, d** for SW480**)** Images of tumors from nude mice at autopsy are presented (left), the tumor volumes were measured at the indicated time points (middle), and the average weight of the xenografted tumors was measured (right). **e** Immunohistochemical staining of Ki67 in xenografted tumors of mice injected with LV-C9–202 or LEV CRC cells. Scale bar, 50 μm. The data are presented as the mean ± s.d. *P < 0.05, ***P < 0.001 by Student’s *t*-test **(a, c, d)** or two-way ANOVA **(b, c, d)**

To verify the effects of CASC9 in CRC cells, HCT-116 and SW480 cells were transduced with CASC9–202-overexpressing lentivirus (LV-C9–202) or control lentivirus (LEV). CASC9 expression was increased by 100- and 1000-fold in LV-C9–202-HCT-116 and LV-C9–202-SW480 cells, respectively (Additional file 6: Figure S5b). As expected, cell proliferation was increased in LV-C9–202 CRC cells as compared to control cells as indicated by colony-formation assays (Fig. 4a). In line herewith, cell viability was promoted by ectopic CASC9–202 expression (Fig. 4b). To determine the effects of CASC9–202 on tumor formation in vivo, LV-C9–202-HCT-116 and LV-C9–202-SW480 cells were subcutaneously injected into nude mice and the mice were sacrificed after 22 and 24 days, respectively. As expected, average tumor volumes and weights were significantly higher in the LV-C9–202 CRC-injected groups than in control animals (Fig. 4c, d). Ki-67 staining revealed that tumors from LV-CASC9–202 cells had more proliferative cells than those in the control group (Fig. 4e). In addition, similar to the effects of CASC9–202, ectopic CASC9–204 expression promoted SW480 cell proliferation in vitro and in vivo (Additional file 7: Figure S6a, b, c, d).

### CASC9 modulates multiple signaling pathways involved in CRC cell growth

**Fig. 5:**
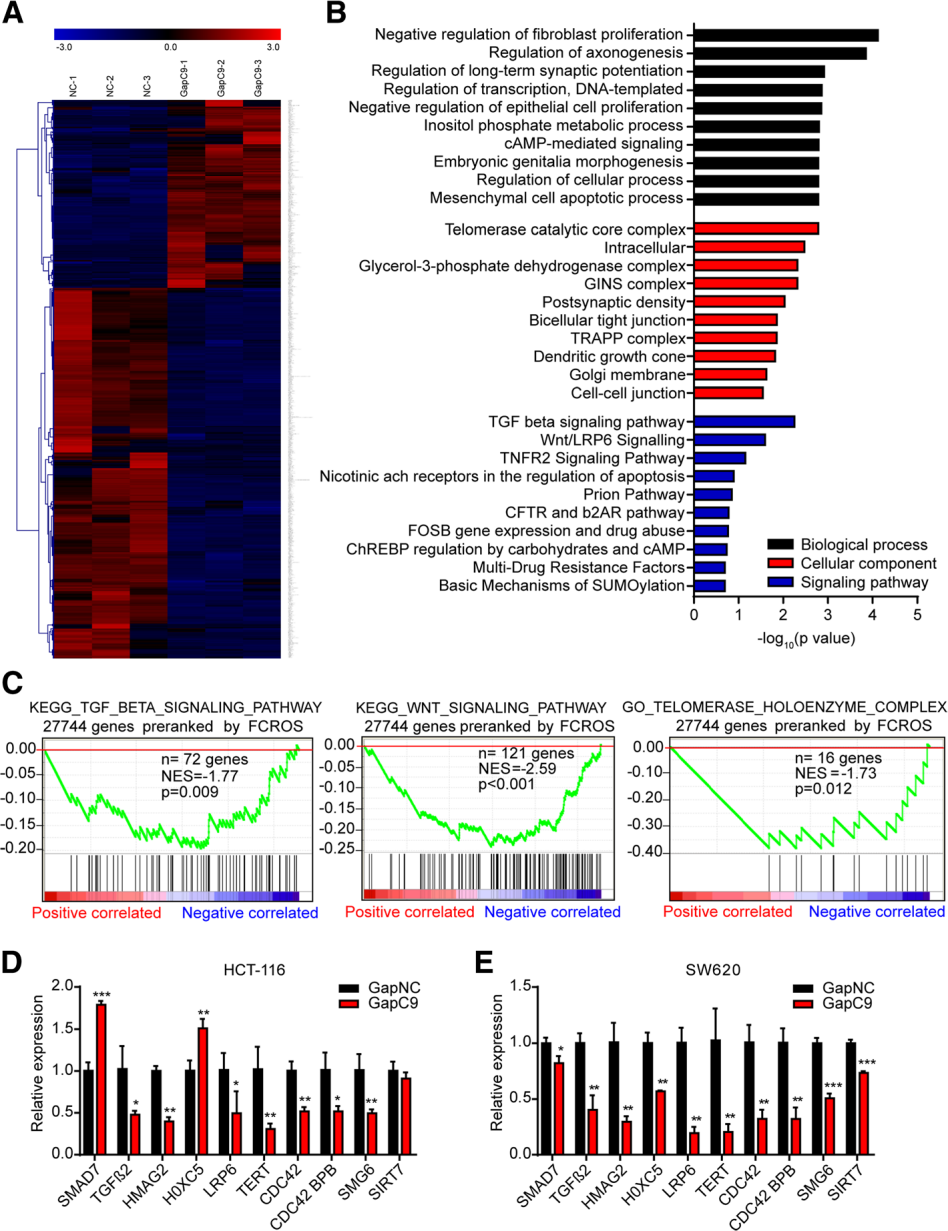
Silencing of CASC9 causes dysregulation of genes involved in cell proliferation in HCT-116 cells. **a** Gene expression profiles of HCT-116 cells transfected with CASC9 GapmeR (GapC9) or control GapmeR (GapNC). Genes (fold change ≥2 or ≤ 0.5, *P* < 0.05) are shaded in blue, black, or red in the heat map to indicate low, intermediate, or high expression, respectively. **b** Functional annotation clustering of genes regulated by CASC9 in HCT-116 cells is shown. The 10 most enriched groups according to GO and BioCarta analysis are ranked based on *P*-values. Black indicates biological process; red, cellular component; and blue, signaling pathway. **c** GSEA of RNA-seq data from CASC9-knockdown HCT-116 cells for TGF-β signaling pathway (left), Wnt signaling pathway (middle), and telomerase holoenzyme complex (right). NES, normalized enrichment score. **d, e** Genes related to the ranked pathways and tumorigenesis were verified by RT-qPCR in HCT-116 **(d)** and SW620 **(e)** cells transfected with GapC9. The data are presented as the mean ± s.d. *P < 0.05, **P < 0.01, ***P < 0.001 by Student’s *t*-test

To obtain a better understanding of the role of CASC9 in promoting CRC cell growth, the effects of CASC9 knockdown with GapmeRs on gene expression were analyzed by RNA-sequencing. In total, 249 significantly upregulated and 491 significantly downregulated genes (fold change ≥2 or ≤ 0.5, *P* < 0.05) were identified in HCT-116 cells transfected with GapC9 for 24 h (Fig. 5a, Additional file 8: Table S2). GO analysis [25, 26] revealed that biological processes related to cell proliferation and apoptosis and the cellular component of telomerase catalytic core complex were enriched among the genes dysregulated by CASC9 knockdown. Interestingly, transforming growth factor-β (TGF-β) signaling and Wnt/LRP6 signaling were the two most enriched pathways based on BioCarta analysis, and may have driven the remarkable changes in biological processes observed (Fig. 5b). GSEA [27, 28] yielded negative enrichment score curves for the TGF-β signaling pathway, Wnt signaling pathway, and telomerase holoenzyme complex (Fig. 5c). Subsequently, genes related to tumorigenesis and the above-mentioned pathways were verified by RT-qPCR. Indeed, most of these genes were commonly regulated by CASC9 both in HCT-116 and SW620 cells (Fig. 5d, e), suggesting that CASC9 may exert effects on CRC oncogenesis, cell growth, apoptosis, and other cellular functions by modulating these signaling pathways in CRC cells.

### CPSF3 interacts with CASC9 and regulates CRC cell growth

**Fig. 6:**
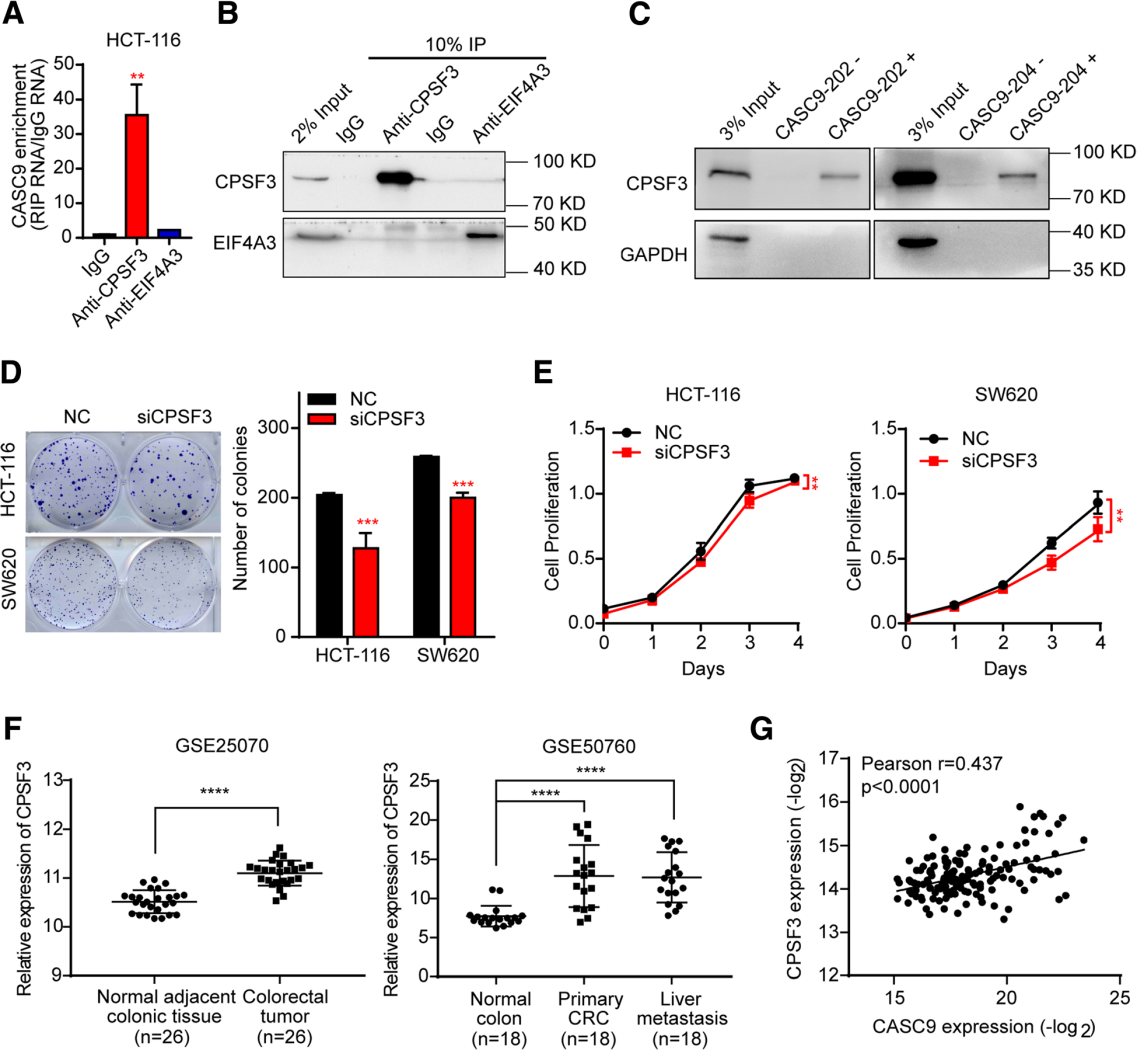
CPSF3 interacts with CASC9 and regulates CRC cell growth. **a** CASC9 RNA was measured by RT-qPCR in IgG control-, CPSF3-, and EIF4A3-RIP samples. **b** Validation of CPSF3- and EIF4A3-immunoprecipitation efficiency by western blotting. **c** Interaction between CASC9–202 or CASC9–204 and CPSF3 was validated by RNA-protein pull-down and western blotting. GAPDH was used as a negative control. **d, e** HCT-116 and SW620 cells were transfected with siRNAs targeting CPSF3 (siCPSF3) or negative control (NC). Cell proliferation was determined by colony-formation assay **(d)** and, at the indicated time points, by MTS assay **(e). f** Scatter plot showing that the expression of CPSF3 was significantly upregulated in CRC tissues as compared to adjacent normal colon tissues in the GSE25070 (*n* = 26, left) and GSE50760 (*n* = 18, right) datasets. **g** Correlation between CASC9 and CPSF3 expression in 155 human CRC tissues. Expression data for CASC9 and CPSF3 were downloaded from the lncRNAtor database. The data are presented as the mean ± s.d. **P < 0.01, ***P < 0.001, ****P < 0.0001 by Student’s *t*-test **(a, d)**, two-way ANOVA **(e),** or Wilcoxon signed-rank test **(f)**

To elucidate the mechanism by which CASC9 regulates CRC cell growth, we attempted to identify its endogenous interaction partners. By analyzing lncRNA–protein interactions using CASC9 (Ensembl ID: ENSG00000249395) in lncRNAtor database [32], CPSF3 and eukaryotic initiation factor 4A-III (EIF4A3) were predicted as potential binding partners of CASC9. Endogenous interactions were verified by RIP assays. CASC9 was indeed significantly enriched (approximately 35-fold) in CPSF3-RIP samples as compared to IgG-RIP samples, whereas CASC9 enrichment in EIF4A3-RIP samples was substantially lower (Fig. 6a, b). We validated the interaction of CASC9 and CPSF3 by RNA-protein pull-down in HCT-116 cells. As expected, CPSF3 was specifically pulled down by CASC9–202 and CASC9–204 affinity complexes, but not by antisense CASC9–202 and antisense CASC9–204, as indicated by western blotting analysis (Fig. 6c). Collectively, these data demonstrated that CASC9 and CPSF3 can form a complex.

To evaluate whether CPSF3 has effects similar to those of CASC9 on CRC tumorigenesis, siRNAs targeting CPSF3 were utilized to suppress its expression, and knockdown efficiency was assessed by both RT-qPCR and western blotting (Additional file 9: Figure S7a, b). As shown in Fig. 6d and e, knockdown of CPSF3 significantly reduced cell proliferation and viability in HCT-116 and SW620 cells. Based on gene expression levels in GEO datasets (GSE25070, GSE50760, GSE44076, and GSE103512), CPSF3 expression was significantly upregulated in CRC tissues as compared to adjacent or non-adjacent normal colon tissues (Fig. 6f, Additional file 10: Figure S8a, b). More importantly, data downloaded from the lncRNAtor database revealed a positive correlation between CASC9 and CPSF3 expression in 155 human CRC tissues (Fig. 6g).

### CASC9 activates TGF-β signaling by interacting with CPSF3 in CRC cells

**Fig. 7:**
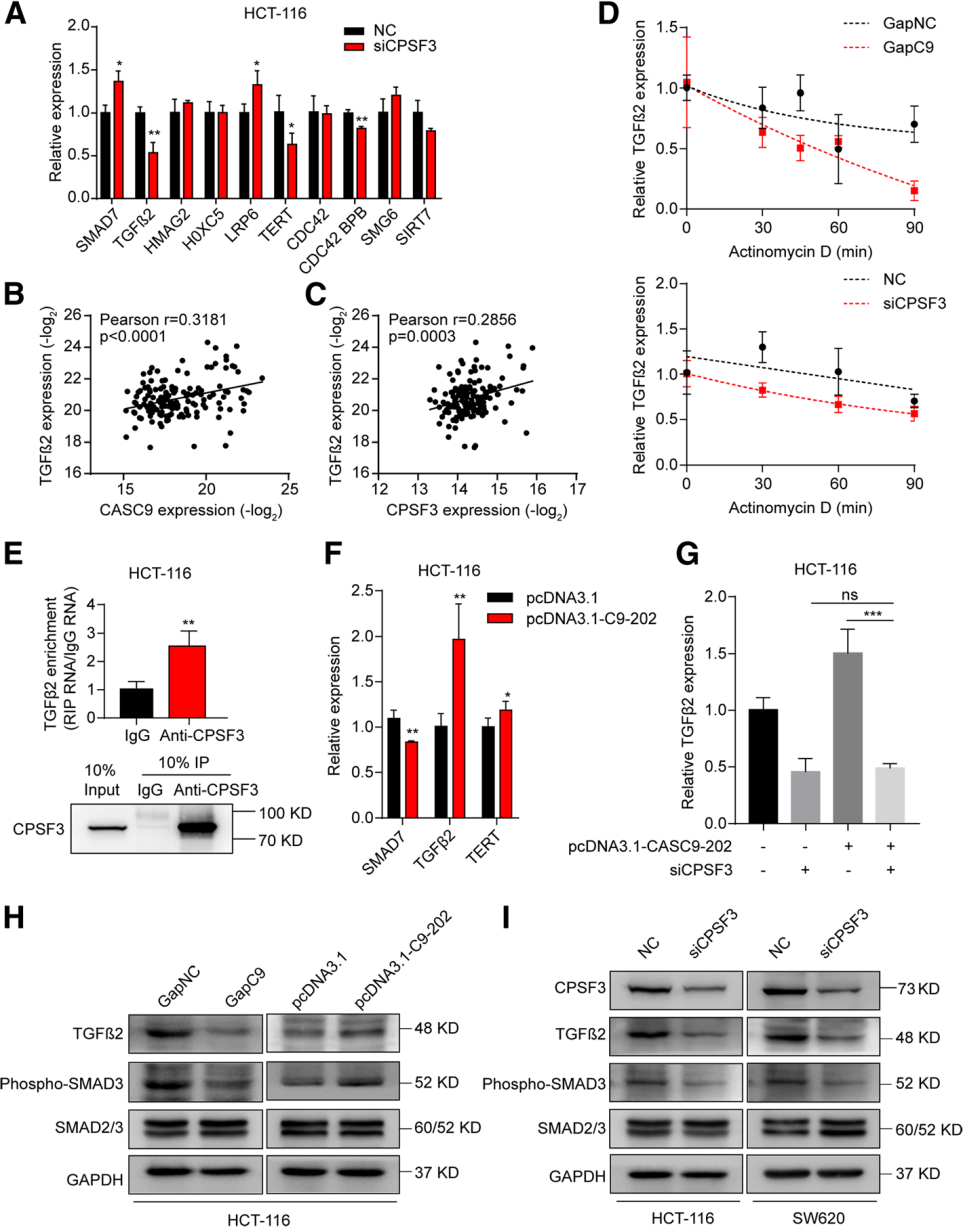
CASC9 and CPSF3 coregulate the expression of TGFβ2. **a** RT-qPCR of selected genes related to the ranked pathways and tumorigenesis in CPSF3-knockdown HCT-116 cells. **b, c** Correlation of CASC9 and TGFβ2 **(b)** and of CPSF3 and TGFβ2 **(c)** expression in 155 human CRC tissues. Expression data for CASC9, CPSF3, and TGFβ2 were downloaded from the lncRNAtor database. **d** The decay of TGFβ2 mRNA was monitored by RT-qPCR in CASC9- or CPSF3-knockdown HCT-116 cells by blocking mRNA synthesis using actinomycin D (ActD, 5 μg/mL) for the indicated time points upon normalization to RNA input levels. **e** RIP revealed that CPSF3 binds TGFβ2 mRNA in HCT-116 cells. Validation of CPSF3-immunoprecipitation efficiency by western blotting. **f** The mRNA levels of SMAD7, TGFβ2, and TERT were determined in HCT-116 cells transfected with pcDNA3.1-CASC9–202. **g** The mRNA levels of TGFβ2 were determined in HCT-116 cells cotransfected with pcDNA3.1-CASC9–202 and siCPSF3. **h** The TGFβ2 protein and phosphorylated SMAD3 levels were determined in CASC9-knockdown or -overexpressing HCT-116 cells. **i** The TGFβ2 protein and phosphorylated SMAD3 levels were determined in CPSF3-knockdown CRC cells. The data are presented as the mean ± s.d. *P < 0.05, **P < 0.01 by Student’s *t*-test **(a, e, f, g)**

The observations that CPSF3 has an effect similar to that of CASC9 in CRC cells and that CPSF3 is a specific interaction partner of CASC9 led us to hypothesize that suppression of CPSF3 would induce regulatory changes similar to those induced by CASC9 silencing. Consistent with the effects of CASC9 silencing, upregulation of SMAD7 and downregulation of TGFβ2, TERT, and CDC42 BPB were observed in CPSF3-knockdown HCT-116 cells (Fig. 7a), indicating that these genes can be regulated directly or indirectly by both CASC9 and CPSF3. However, LRP6 expression was differentially affected by CASC9 and CPSF3 knockdown (Fig. 7a). In addition, positive correlations were observed between TGFβ2 and CASC9 or CPSF3 expression in human CRC tissues (Fig. 7b, c, Additional file 10: Figure S8c, d, e, f). Furthermore, TGFβ2 mRNA decayed more rapidly upon silencing of CASC9 or CPSF3, indicating that both of them interfere with TGFβ2 mRNA turnover (Fig. 7d). Moreover, RIP revealed that CPSF3 binds TGFβ2 mRNA (Fig. 7e). Ectopic CASC9–202 expression led to increased TGFβ2 mRNA levels (Fig. 7f), which were restored by knockdown of CPSF3 (Fig. 7g). Accordingly, TGFβ2 protein and phosphorylated SMAD3 levels were reduced in CASC9-knockdown cells, whereas their levels were increased by ectopic CASC9 expression (Fig. 7h). In line herewith, TGFβ2 protein and phosphorylated SMAD3 levels were reduced in CPSF3-knockdown cells (Fig. 7i). Collectively, these data provided evidence supporting the positive regulation of TGFβ2 expression by the CASC9/CPSF3 complex in CRC cells.

## Discussion

In this study, through combined bioinformatics and experimental analyses, we discovered that CASC9 is mainly expressed in normal colon and bladder tissues. Furthermore, CRC tissues exhibited increased CASC9 expression, which was correlated with advanced TNM stage, and higher CASC9 levels were associated with poor patient outcomes. In addition, CASC9 promoted cell growth and decreased apoptosis in vitro and was required for tumor formation in nude mice in vivo*.* These data provide strong evidence supporting an oncogenic role of CASC9 in CRC, in accordance with previous reports of CASC9 functioning as an oncogene in several other cancers, including hepatocellular carcinoma, esophageal squamous cell carcinoma, gastric cancer, ovarian cancer, glioma, and breast cancer [33–39]. These results demonstrate that higher levels of CASC9 are related to more aggressive disease. Our study is the first to demonstrate that CASC9 acts as an oncogene in CRC.

In our bioinformatics analysis of TCLA and TANRIC [29] data, two additional upregulated lncRNAs with significant prognostic value were identified; however, increased expression of these lncRNAs was associated favorable prognosis. Among them, ZFAS1 is dysregulated and acts as either an oncogene or a tumor suppressor in various human malignancies [40, 41]. In CRC, recent studies have shown that ZFAS1 acts as an oncogene during tumorigenesis [42, 43]. LINC01123 was reported to be a novel and important prognostic factor in head and neck squamous cell carcinoma [44]. Apart from these upregulated lncRNAs, we identified three lncRNAs that were downregulated in CRC and were associated with poor patient outcomes; the roles of these lncRNAs remain to be elucidated.

In our study, CASC9–202 and − 204 were the dominant variants generated from the CASC9 gene in CRC cells, and they were localized in both the nucleus and the cytoplasm, with variable expression levels in different CRC cells. Similar observations were reported in esophageal squamous cancer cells [37]. In contrast, CASC9 was detected mainly in the cytoplasm of hepatocellular carcinoma cells [34] and in the nucleus of lung adenocarcinoma cells [45]. These differences may be explained by cell-specific differences and suggest diverse functions of CASC9 in tumorigenesis. Cytoplasmic lncRNAs are more effectively suppressed by RNA interference, whereas antisense oligonucleotide-directed RNA degradation is effective in both the nuclear and cytoplasmic compartments [31, 46]. There is no evidence that the previously reported CASC9 siRNA-mediated phenotype was specific to the loss of nuclear CASC9. By contrast, this has been taken into account in our study. To explore the biological role of CASC9 in CRC cells further, we knocked down CASC9 in CRC cells using two strategies, i.e., via siRNA and via antisense oligonucleotides. As expected, silencing of CASC9 by siRNA or GapmeR inhibited cell proliferation and induced cell apoptosis in vitro; the knockdown efficiency and biological effects of CASC9 knockdown by GapmeR were more effective than those induced by siRNA. This was consistent with the localization of CASC9 in CRC cells. Conversely, ectopic overexpression of the CASC9–202 or − 204 variants promoted CRC cell growth in vitro and tumor formation in vivo*,* suggesting that the different variants play similar biological roles.

Increasing evidence demonstrates that lncRNAs typically exert their biological functions through the actions of their targeted proteins [47]. For example, LINC01138 specifically binds with PRMT5 in hepatocellular carcinoma cells, and this association may be a potential therapeutic target in hepatocellular carcinoma [48]. SNHG5 directly binds and regulates SPATS2, and this interaction is important for promoting CRC cell survival [49]. In this report, the interaction between CASC9 and CPSF3 was established in CRC cells by RIP and RNA-protein pull–down assays. CPSF3 is an endonuclease that has multiple functions in several biological processes. It is required for the cleavage of mRNAs and is involved in the generation of the 3′ ends of histone mRNAs [50, 51]. Moreover, CPSF3 and the spliceosome-associated ISY1 are responsible for pro-miRNA biogenesis and the expression of most miRNAs within the miR-17-92 cluster [52]. The miR-17-92 miRNA cluster is upregulated and plays multifaceted roles in several malignancies [53, 54]. In CRC, this cluster has been shown to augment tumor angiogenesis by repressing TSP1 and CTGF [55], suggesting that CPSF3 may also be involved in these processes. Indeed, inactivation of CPSF3 is required for the induction of cell apoptosis by the tumor suppressor CSR1 [56]. In our study, knockdown of CPSF3 inhibited cell growth in a manner similar to that of CASC9 silencing in CRC cells. In addition, CPSF3 was significantly upregulated in CRC tissues as compared to normal colon tissues from several independent cohorts. More importantly, CASC9 and CPSF3 expression was significantly positively correlated in CRC tissues. Collectively, our data support the idea that CASC9 binds to CPSF3 and that they potentially regulate downstream pathways together.

Using RNA-seq analysis, we found that the silencing of CASC9 regulates several genes involved in cell proliferation and apoptosis. Pathway analysis revealed that the dysregulated genes were significantly linked to several signal transduction pathways, including the TGF-β, Wnt/LRP6, and TERT signaling pathways, which are commonly activated in CRC progression [57, 58]. The dysregulation of key genes related to the above pathways, including TGFβ2, LRP6, and TERT, was confirmed by RT-qPCR in CRC cells, although the expression of SMAD7 and HOXC5 was differentially regulated between CASC9-silenced HCT-116 and SW620 cells. This phenomenon may be explained by cell-specific differences. In fact, CRC cell lines are classified according to their genetic and epigenetic molecular phenotypes, including microsatellite instability and the CpG island methylator phenotype [59]. Upon CPSF3 knockdown, TGFβ2 and TERT were also significantly decreased, whereas SMAD7 was significantly increased, with the degree of regulation being more moderate than, but similar to that induced by CASC9 silencing. Other evidence also supports the coregulation of the TGF-β/SMAD pathways by CASC9 and CPSF3. First, TGFβ2 mRNA was destabilized after knockdown of CASC9 or CPSF3. Second, ectopic CASC9–202 expression increased TGFβ2 mRNA levels, which were restored by knockdown of CPSF3. Third, significant correlations between TGFβ2 and CASC9 or CPSF3 expression were found in CRC tissues. In addition, we found that LRP6, a key component of the Wnt pathway, was regulated by CASC9, but not by CPSF3, suggesting that CASC9 has other interaction partners, which remain to be identified.

## Conclusions

Our data indicate that CASC9 acts as an oncogene in CRC and significantly promotes CRC cell growth in vitro and in vivo. More specifically, CASC9 interacts with CPSF3 and exerts its oncogenic activity by activating TGF-β signaling and TERT complex function in CRC cells (Additional file 11: Figure S9). Our findings suggest that manipulating the expression of CASC9 or its partner proteins may be a promising therapeutic approach in CRC. However, detailed roles of the different CASC9 variants and their upstream factors remain unknown and require further in-depth study.

## Additional files

Additional file 1: **Table S1.** Oligonucleotides used for RT-qPCR, plasmid construction, RNA pull-down, siRNA, and GapmeR. (DOCX 25 kb)

Additional file 2: **Figure S1.** Identification of dysregulated lncRNAs with significant prognostic value in COAD. (A) Schematic representation of approach used to identify downregulated lncRNAs in COAD. (B) Three upregulated lncRNAs had significant prognostic value in COAD. (C) Five downregulated lncRNAs had significant prognostic value in COAD. (TIF 1815 kb)

Additional file 3: **Figure S2.** CASC9 gene expression in 53 tissues from GTEx RNA-seq data of 8555 samples (570 donors). (TIF 727 kb)

Additional file 4: **Figure S3.** Knockdown of CASC9 by siRNA inhibits proliferation of SW620 cells*.* SW620 cells were transfected with siRNA targeting CASC9 (siC9–1 and siC9–2) or negative control (NC). (A) The siRNA knockdown efficiency of CASC9 was determined by RT-qPCR. (B, C) Cell proliferation was determined by colony formation assay (B) and, at the indicated time points, by MTS assay (C). (D) Cell cycle was determined by flow cytometry. Quantification of the percentages of cells in G0/G1, S, and G2/M phases (top) and representative graphs of raw data (bottom) are shown. The data are presented as mean ± s.d. *P < 0.05, **P < 0.01, ***P < 0.001, ****P < 0.0001 by Student’s *t*-test (A, B, D) or two-way ANOVA (C). (TIF 1673 kb)

Additional file 5: **Figure S4.** Knockdown efficiency of CASC9 by Antisense LNA GapmeR or siRNA in CRC cells. (A) RT-qPCR analysis of relative CASC9 levels in HCT-116 and SW620 cells transfected with Antisense LNA GapmeRs targeting CASC9 (GapC9) or control GapmeRs (GapNC). (B) Relative CASC9 levels following biochemical fractionation of HCT-116 and SW620 cells transfected with siRNAs (left) or GapmeR (right). (C) Representative graphs of raw data for cell cycle analysis by flow cytometry (related to Fig. 2c). The data are presented as mean ± s.d. *P < 0.05, **P < 0.01, ***P < 0.001 by Student’s *t*-test. (TIF 1029 kb)

Additional file 6: **Figure S5.** Knockdown or overexpression efficiency of CASC9 in stable CRC cell lines. (A) RT-qPCR analysis of relative CASC9 levels in HCT-116 and SW620 cells transduced with shCASC9 lentivirus (shC9–1 and shC9–2) or control lentivirus (shCON). (B) RT-qPCR analysis of relative CASC9 levels in HCT-116 and SW480 cells transduced with CASC9–202-overexpressing lentivirus (LV-C9–202) or control lentivirus (LEV). The data are presented as mean ± s.d. *P < 0.05, **P < 0.01, ****P < 0.0001 by Student’s *t*-test. (TIF 438 kb)

Additional file 7: **Figure S6.** Ectopic expression of CASC9–204 promotes proliferation of CRC cells in vitro*.* SW480 cells were transduced with CASC9–204-overexpressing lentivirus (LV-C9–204) or control lentivirus (LEV). (A) RT-qPCR analysis of relative CASC9 levels in LV-C9–204-SW480 cells. (B, C) Cell proliferation was determined by colony formation assay (B) and, at the indicated time points, by MTS assay (C). (D) Subcutaneous xenografts of SW480 cells transduced with LV-C9–204 or LEV (*n* = 5). Images of the tumors at autopsy from nude mice are presented (left), the tumor volumes were measured at the indicated time points (middle), and the average weight of the xenografted tumors was measured (right). The data are presented as mean ± s.d. **P < 0.01, ****P < 0.0001 by Student’s *t*-test (A, B, D) or two-way ANOVA (C, D). (TIF 2037 kb)

Additional file 8: **Table S2.** Gene differential expression in HCT-116/GapC9 and HCT-116/GapNC cells. (DOCX 136 kb)

Additional file 9: **Figure S7.** Knockdown efficiency of CPSF3 was determined in HCT-116 and SW620 cells transfected with siRNA targeting CPSF3 (siCPSF3) or negative control (NC). (A) RT-qPCR analysis of relative CPSF3 mRNA levels. (B) Western blotting analysis of protein levels of CPSF3. The data are presented as mean ± s.d. *P < 0.05, **P < 0.01 by Student’s *t*-test. (TIF 537 kb)

Additional file 10: **Figure S8.** CPSF3 was upregulated in CRC tissues. (A, B) Scatter plot showing that the expression of CPSF3 was significantly upregulated in CRC tissues compared with levels in adjacent or non-adjacent normal colon tissues in the GSE44076 (A) and GSE103512 (B) datasets. (C, D, E, F) Correlations of CASC9 and TERT (C, left), of CPSF3 and TERT (C, right), of CASC9 and SMAD7 (D, left), of CPSF3 and SMAD7 (D, right), CASC9 and LRP6 (E, left), of CPSF3 and LRP6 (E, right), and CASC9 and CDC42 BPB (F, left), of CPSF3 and CDC42 BPB (F, right), expression in 155 human CRC tissues. Expression data for CASC9, CPSF3, SMAD7, TERT, LRP6, and CDC42 BPB were downloaded from the lncRNAtor database. The data are presented as mean ± s.d. ****P < 0.0001 by Wilcoxon signed-rank test or Mann-Whitney *U*-test. (TIF 1526 kb)

Additional file 11: **Figure S9.** Schematic diagram of lncRNA CASC9 interacting with CPSF3 to co-regulate genes linked to TGF-β signaling and TERT complex function and its roles in CRC tumorigenesis. (TIF 1043 kb)

## Data Availability

The datasets during and/or analyzed in the current study are available from the corresponding author on reasonable request.

## Abbreviations

ANOVA: Analysis of variance
ATCC: American type culture collection
CASC9: Cancer susceptibility candidate 9
CCAT1: Colon cancer-associated transcript-1
COAD: Colon adenocarcinoma
CPSF3: Cleavage and polyadenylation specificity factor subunit 3
CRC: Colorectal cancer
GO: Gene ontology
GSEA: Gene set enrichment analysis
lncRNA: Long non-coding RNA
MTS: 3-(4,5-dimethylthiazol-2-yl)-5-(3-carboxymethoxyphenyl)-2-(4-sulfophenyl)-2H-tetrazolium
PBS: Phosphate-buffered saline
PI: Propidium iodide
RIP assay: RNA immunoprecipitation assay
RT-qPCR: Reverse transcription quantitative PCR
s.d.: Standard deviation
siRNA: Small interfering RNA
TANRIC: The Atlas of Noncoding RNAs in Cancer
TCLA: The Cancer LncRNome Atlas
