# Supplementary material for: LncRNA CASC9 interacts with CPSF3 to regulate TGF-β signaling in colorectal cancer
Source: J Exp Clin Cancer Res. 2019 Jun 11;38:249. doi: 10.1186/s13046-019-1263-3 (PMC6560732; doi:10.1186/s13046-019-1263-3)
Supplement: Supplementary file 1 — Table S1. Oligonucleotides used for RT-qPCR, plasmid construction, RNA pull-down, siRNA, and GapmeR. (DOCX 25 kb) [file 13046_2019_1263_MOESM1_ESM.docx]

| **Additional file 1: Table S1. Oligonucleotides used for RT-qPCR, plasmid construction, RNA pull-down, siRNA, and GapmeR.** | | |
| --- | --- | --- |
| **Name** | **Forward** | **Reverse** |
| **Oligonucleotides used for RT-qPCR** |  |  |
| CASC9-Total | 5’-TTGGTCAGCCACATTCATGGT-3’ | 5’-AGTGCCAATGACTCTCCAGC-3’ |
| CASC9‐201 | 5’-TCCTGGATGGTCTTGGTGTT-3’ | 5’-ACCATGAATGTGGCTGACCA-3’ |
| CASC9‐202 | 5’-GCTGTAGACCGGAGCTGTTC-3’ | 5’-ACACCATGAATGTGGCTGAC-3’ |
| CASC9‐203 | 5’-CAATCAGCGAGACTCCGTGG-3’ | 5’-CCATGAATGTGGCTGACCAA-3’ |
| CASC9‐204 | 5’-CATCTTGGCTCCTCCGAATGTG-3’ | 5’-TCTTGCCAGGTGTTGTTCTGC-3’ |
| CPSF3 | 5’-GCACGTTTACAGCAAGAGGTTGG-3’ | 5’-AAGGTTGGCAGTTTTCCCGTCC-3’ |
| HOXC5 | 5’-GACCAAACTGCACATGAGCCAC-3’ | 5’-GTTGTTGGCGATCTCTATGCGC-3’ |
| TERT | 5’-CCGATTGTGAACATGGACTACG-3’ | 5’-CACGCTGAACAGTGCCTTC-3’ |
| LRP6 | 5’-CAGTTGGAGTGGTGCTGAAAGG-3’ | 5’-CCATCCAAAGCAGCCCGTTCAA-3’ |
| HMGA2 | 5’-GAAGCCACTGGAGAAAAACGGC-3’ | 5’-GGCAGACTCTTGTGAGGATGTC-3’ |
| CDC42 | 5’-TGACAGATTACGACCGCTGAGTT-3’ | 5’-GGAGTCTTTGGACAGTGGTGAG-3’ |
| SKI | 5’-CCTTCCGAAAAGGACAAGCCGT-3’ | 5’-GCTCTTTCTCACTCGCTGACAC-3’ |
| SIRT7 | 5’-TGGAGTGTGGACACTGCTTCAG-3’ | 5’-CCGTCACAGTTCTGAGACACCA-3’ |
| CDC42BPB | 5’-GCATCGGAACAGCCTACAAAGG-3’ | 5’-AGACTTGGCTCGCAATGACACC-3’ |
| SMAD7 | 5’-TGTCCAGATGCTGTGCCTTCCT-3’ | 5’-CTCGTCTTCTCCTCCCAGTATG-3’ |
| TGFB2 | 5’-AAGAAGCGTGCTTTGGATGCGG-3’ | 5’-ATGCTCCAGCACAGAAGTTGGC-3’ |
| SKIL | 5’-GCAGGAAGGTGACCATGTTTCTC-3’ | 5’-TACTTGCCTCTGTCTTTGTGAGC-3’ |
| SMG6 | 5’-GATGGTCTTGCCATTCGCAGCA-3’ | 5’-TCGCTGTATCACTGGCTTGCTC-3’ |
| β-actin | 5’-GAGCTACGAGCTGCCTGACG-3’ | 5’-GTAGTTTCGTGGATGCCACAG-3’ |
| GAPDH | 5’-ACCCACTCCTCCACCTTTG-3’ | 5’-CACCACCCTGTTGCTGTAG-3’ |
| NEAT1 | 5’-GCTGGACCTTTCATGTAACGGG-3’ | 5’-TGAACTCTGCCGGTACAGGGAA-3’ |
| **Oligonucleotides used for plasmid construction** | |  |
| LV-CASC9-202 | 5’-CGGTGAATTCCTCGAGCCGGTACCTCAGATGGAAATG-3’ | 5’-CCGCGGCCGCTCTAGACCACTGTCTTCTGGGCTTAAT-3’ |
| LV-CASC9-204 | 5’-CGGTGAATTCCTCGAGATGCAGAAATCACCCGTCTT-3’ | 5’-CCGCGGCCGCTCTAGATGGAATCCAGTTGAAAGGTTCT-3’ |
| shCASC9-1 | 5’-GGAAAGCAATGGAAGCATGTATTCTCGAGAATACATGCTTCCATTGCTTTTTTTTG-3’ | 5’-AATTCAAAAAAAAGCAATGGAAGCATGTATTCTCGAGAATACATGCTTCCATTGCTTT-3’ |
| shCASC9-2 | 5’-CCGGAAGCCTGTGATAGCAGAACAACTCGAGTTGTTCTGCTATCACAGGCTTTTTTTG-3’ | 5’-AATTCAAAAAAAGCCTGTGATAGCAGAACAACTCGAGTTGTTCTGCTATCACAGGCTT-3’ |
| **Oligonucleotides used for RNA pull-down** | |  |
| CASC9-202 | 5’-TAATACGACTCACTATAGGTCTGGCACTCCCTAGTGAG-3’ | 5’-TAGCATTTCTGTTAACTTGGGGT-3’ |
| Anti-CASC9-202 | 5’-GTCTGGCACTCCCTAGTGAG-3’ | 5’-TAATACGACTCACTATAGTAGCATTTCTGTTAACTTGGGGT-3’ |
| CASC9-204 | 5’-TAATACGACTCACTATAGCTCAGATGGAAATGCAGAAA-3’ | 5’-TGACATATTAAAGTTGGAATCC-3’ |
| Anti-CASC9-204 | 5’-CTCAGATGGAAATGCAGAAA-3’ | 5’-TAATACGACTCACTATAGTGACATATTAAAGTTGGAATCC-3’ |
| **Oligonucleotides used for siRNA** |  |  |
| siCASC9-1 | 5’-CAGCAAAGCAAUGGAAGCAUGUAUU-3’ | 5’-AAUACAUGCUUCCAUUGCUUUGCUG-3’ |
| siCASC9-2 | 5’-GCCUGUGAUAGCAGAACAATT-3’ | 5’-UUGUUCUGCUAUCACAGGCTT-3’ |
| siCPSF3 | 5’-GCAGACGACAUGCUGUAUATT-3’ | 5’-UAUACAGCAUGUCGUCUGCTT-3’ |
| siCON | 5’-UUCUCCGAACGUGUCACGUTT-3’ | 5’-ACGUGACACGUUCGGAGAATT-3’ |
| **Oligonucleotides used for GapmeR ("*" indicated the phosphorothioate backbone modifications. The position of the LNA modifications is not shown.)** | | |
| GapmeR NC | 5’-G*C*T*C*C*C*T*T*C*A*A*T*C*C*A*A-3’ | Negative control B Cat.no. 339515 LG00000001-DDA (QIAGEN) |
| GapmeR CASC9 | 5’-G*G*C*T*G*A*C*C*A*A*T*C*A*C*T*T-3’ | Cat.no. 339511 LG00201872-DDA (QIAGEN) |
