# Supplementary figures and images for: LncRNA CASC9 interacts with CPSF3 to regulate TGF-β signaling in colorectal cancer
Source: J Exp Clin Cancer Res. 2019 Jun 11;38:249. doi: 10.1186/s13046-019-1263-3 (PMC6560732; doi:10.1186/s13046-019-1263-3)

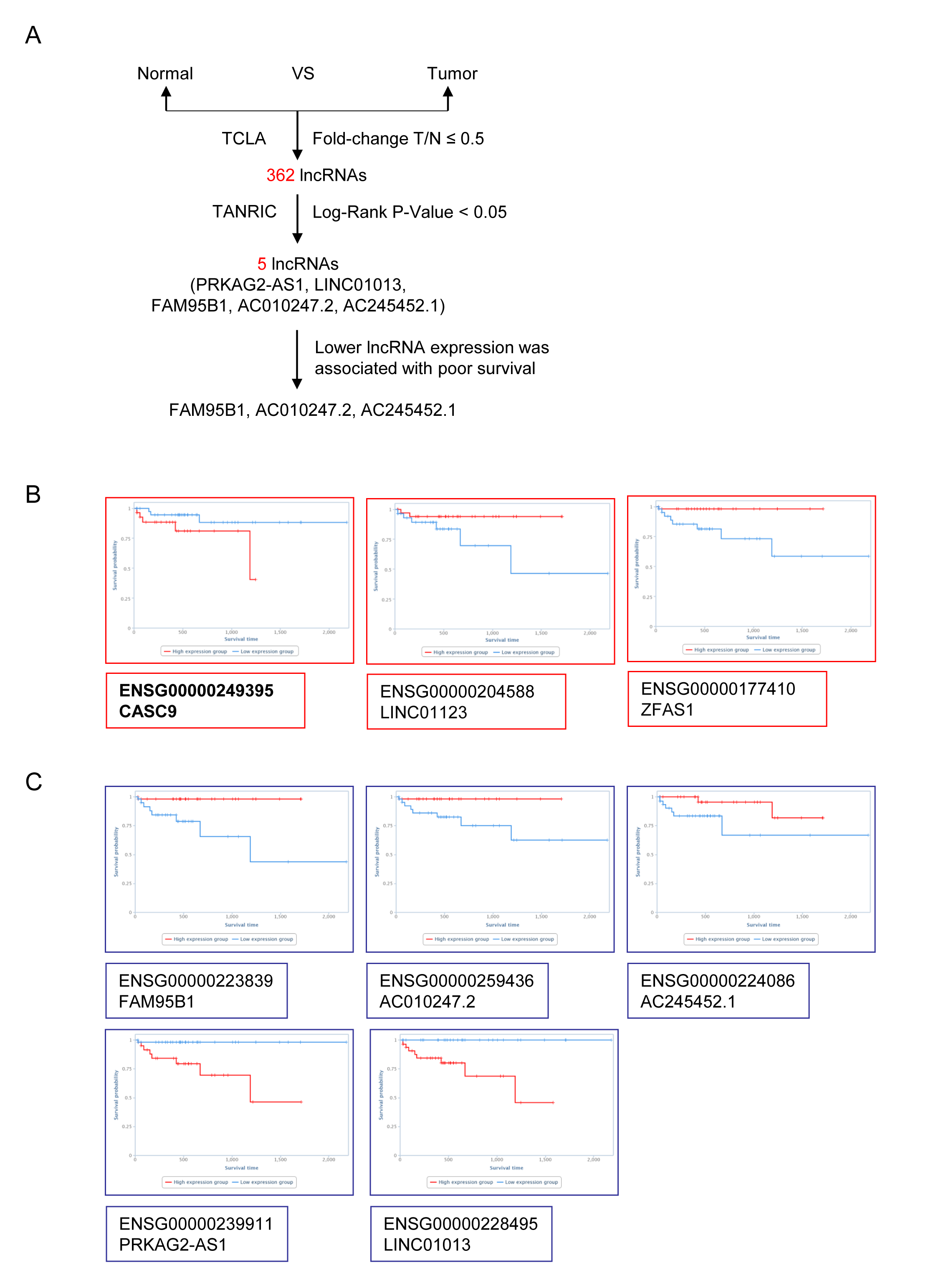

Supplement: Supplementary file 2 — Figure S1. Identification of dysregulated lncRNAs with significant prognostic value in COAD. (A) Schematic representation of approach used to identify downregulated lncRNAs in COAD. (B) Three upregulated lncRNAs had significant prognostic value in COAD. (C) Five downregulated lncRNAs had significant prognostic value in COAD. (TIF 1815 kb) [file 13046_2019_1263_MOESM2_ESM.tif]

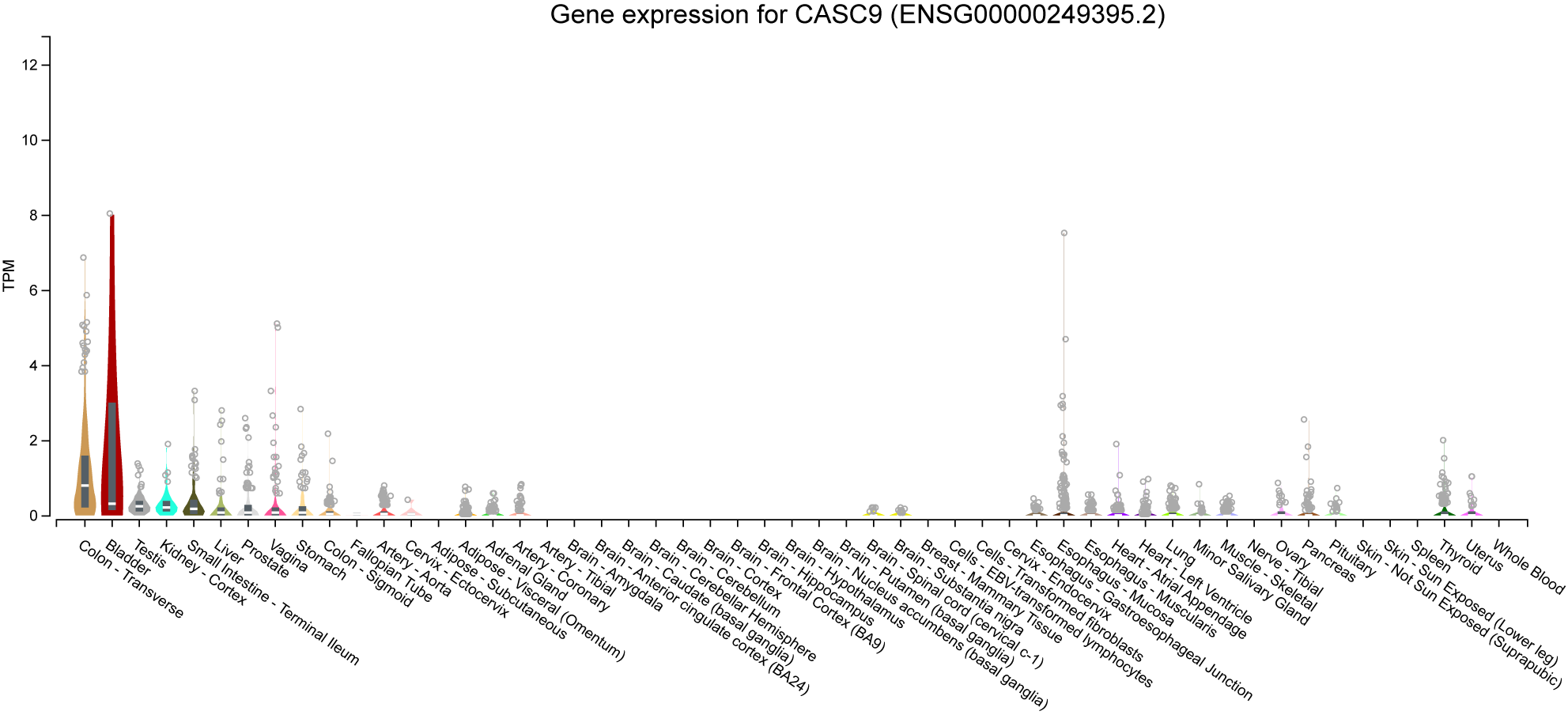

Supplement: Supplementary file 3 — Figure S2. CASC9 gene expression in 53 tissues from GTEx RNA-seq data of 8555 samples (570 donors). (TIF 727 kb) [file 13046_2019_1263_MOESM3_ESM.tif]

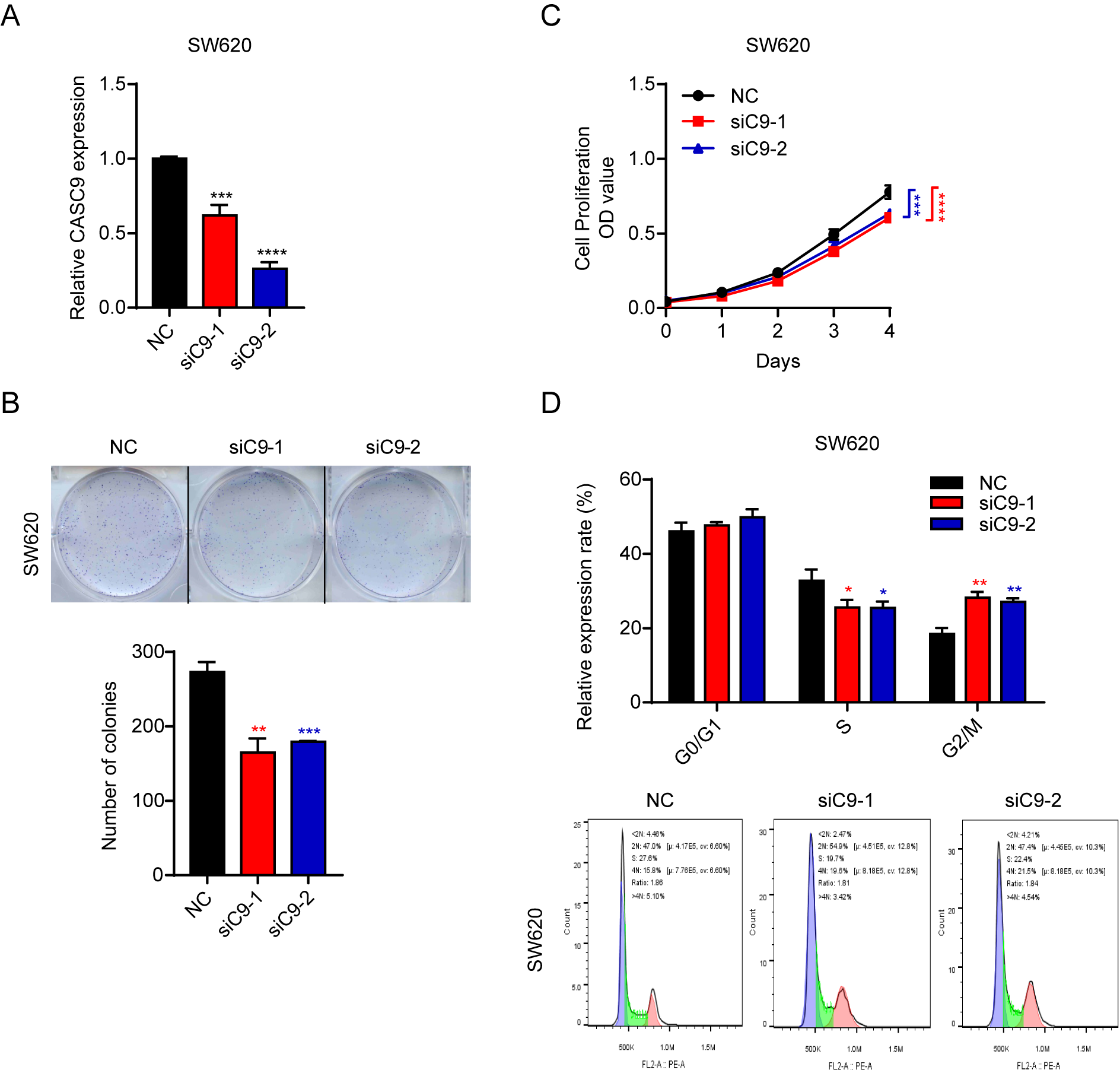

Supplement: Supplementary file 4 — Figure S3. Knockdown of CASC9 by siRNA inhibits proliferation of SW620 cells. SW620 cells were transfected with siRNA targeting CASC9 (siC9–1 and siC9–2) or negative control (NC). (A) The siRNA knockdown efficiency of CASC9 was determined by RT-qPCR. (B, C) Cell proliferation was determined by colony formation assay (B) and, at the indicated time points, by MTS assay (C). (D) Cell cycle was determined by flow cytometry. Quantification of the percentages of cells in G0/G1, S, and G2/M phases (top) and representative graphs of raw data (bottom) are shown. The data are presented as mean ± s.d. *P < 0.05, **P < 0.01, ***P < 0.001, ****P < 0.0001 by Student’s t-test (A, B, D) or two-way ANOVA (C). (TIF 1673 kb) [file 13046_2019_1263_MOESM4_ESM.tif]

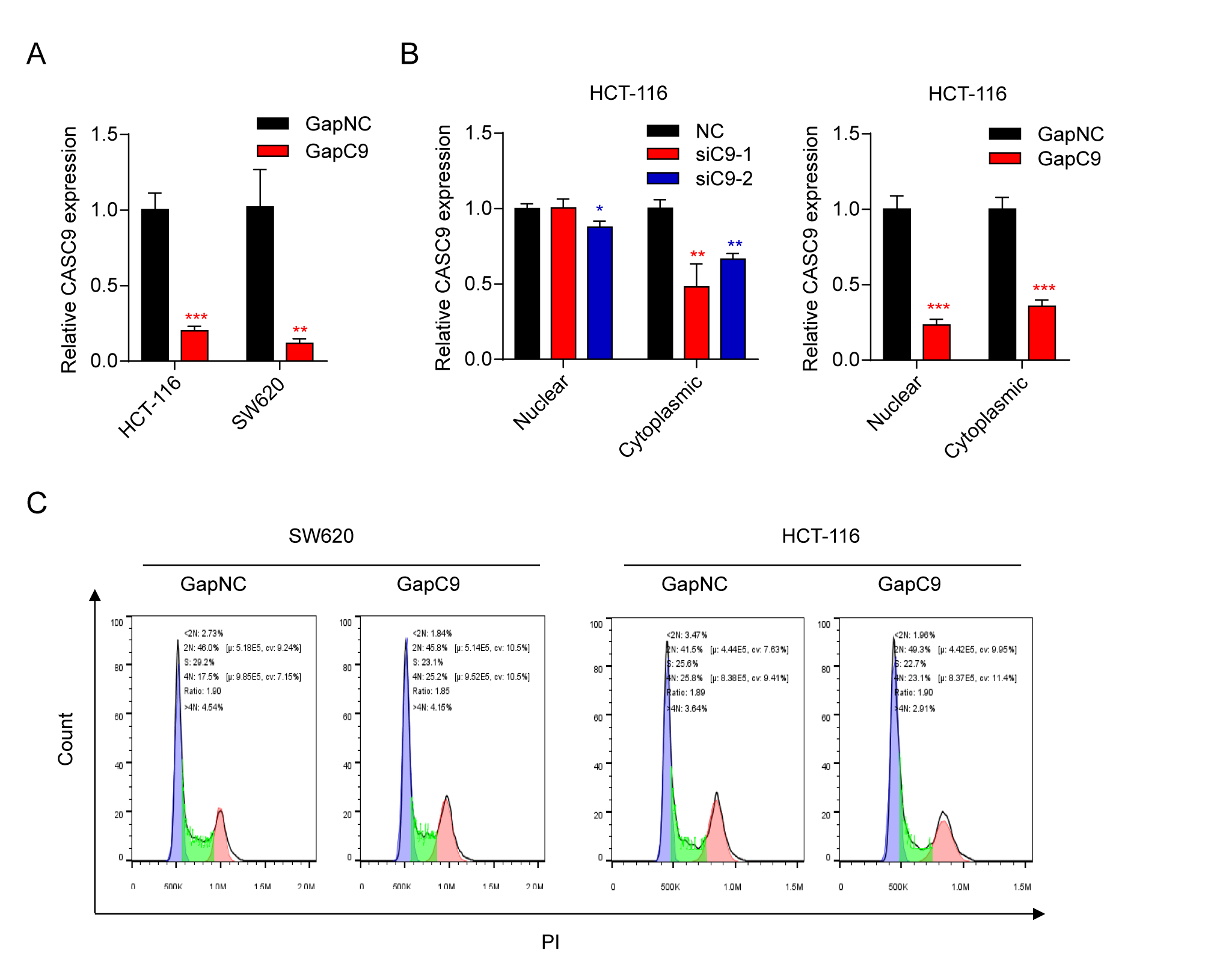

Supplement: Supplementary file 5 — Figure S4. Knockdown efficiency of CASC9 by Antisense LNA GapmeR or siRNA in CRC cells. (A) RT-qPCR analysis of relative CASC9 levels in HCT-116 and SW620 cells transfected with Antisense LNA GapmeRs targeting CASC9 (GapC9) or control GapmeRs (GapNC). (B) Relative CASC9 levels following biochemical fractionation of HCT-116 and SW620 cells transfected with siRNAs (left) or GapmeR (right). (C) Representative graphs of raw data for cell cycle analysis by flow cytometry (related to Fig. 2c). The data are presented as mean ± s.d. *P < 0.05, **P < 0.01, ***P < 0.001 by Student’s t-test. (TIF 1029 kb) [file 13046_2019_1263_MOESM5_ESM.tif]

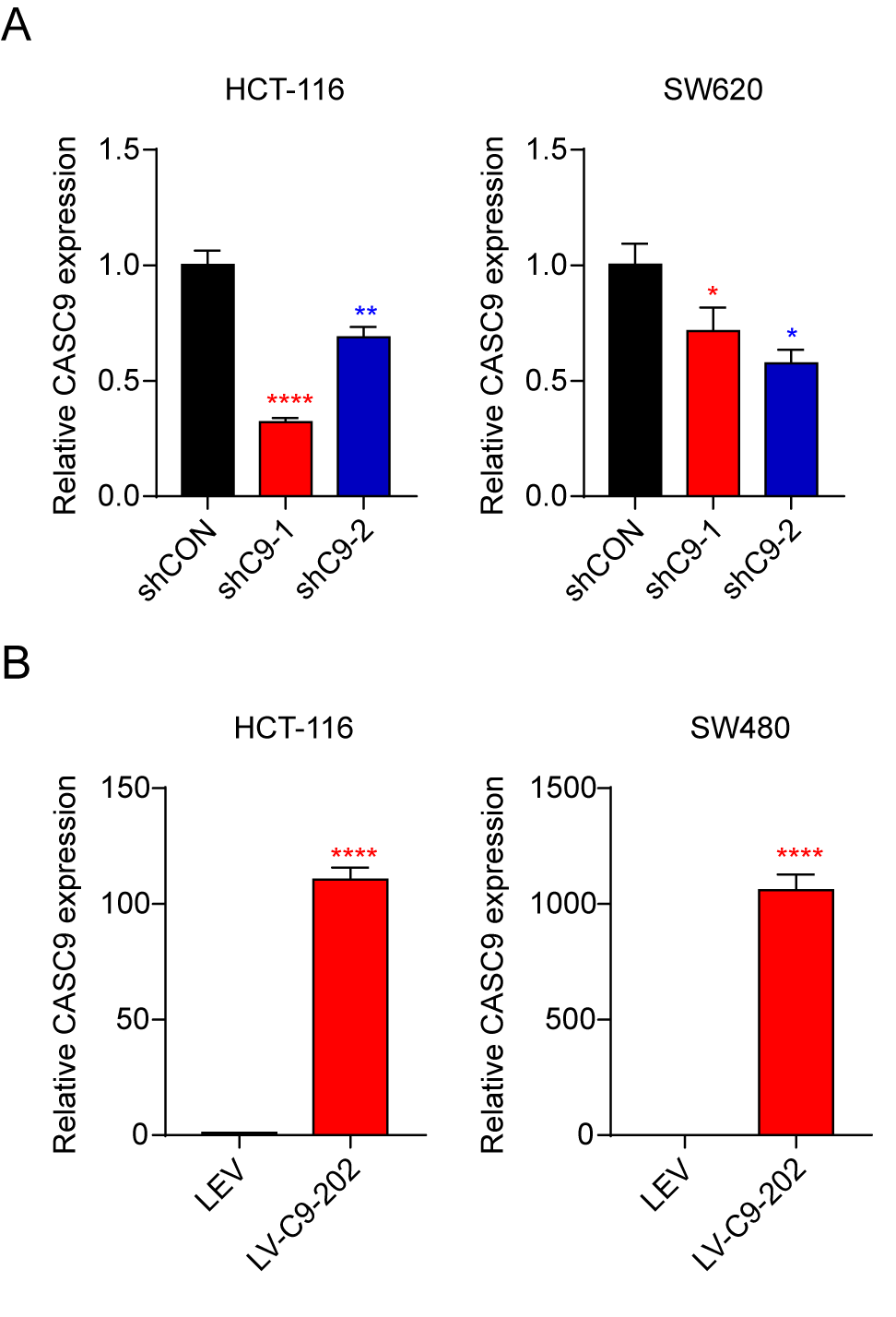

Supplement: Supplementary file 6 — Figure S5. Knockdown or overexpression efficiency of CASC9 in stable CRC cell lines. (A) RT-qPCR analysis of relative CASC9 levels in HCT-116 and SW620 cells transduced with shCASC9 lentivirus (shC9–1 and shC9–2) or control lentivirus (shCON). (B) RT-qPCR analysis of relative CASC9 levels in HCT-116 and SW480 cells transduced with CASC9–202-overexpressing lentivirus (LV-C9–202) or control lentivirus (LEV). The data are presented as mean ± s.d. *P < 0.05, **P < 0.01, ****P < 0.0001 by Student’s t-test. (TIF 438 kb) [file 13046_2019_1263_MOESM6_ESM.tif]

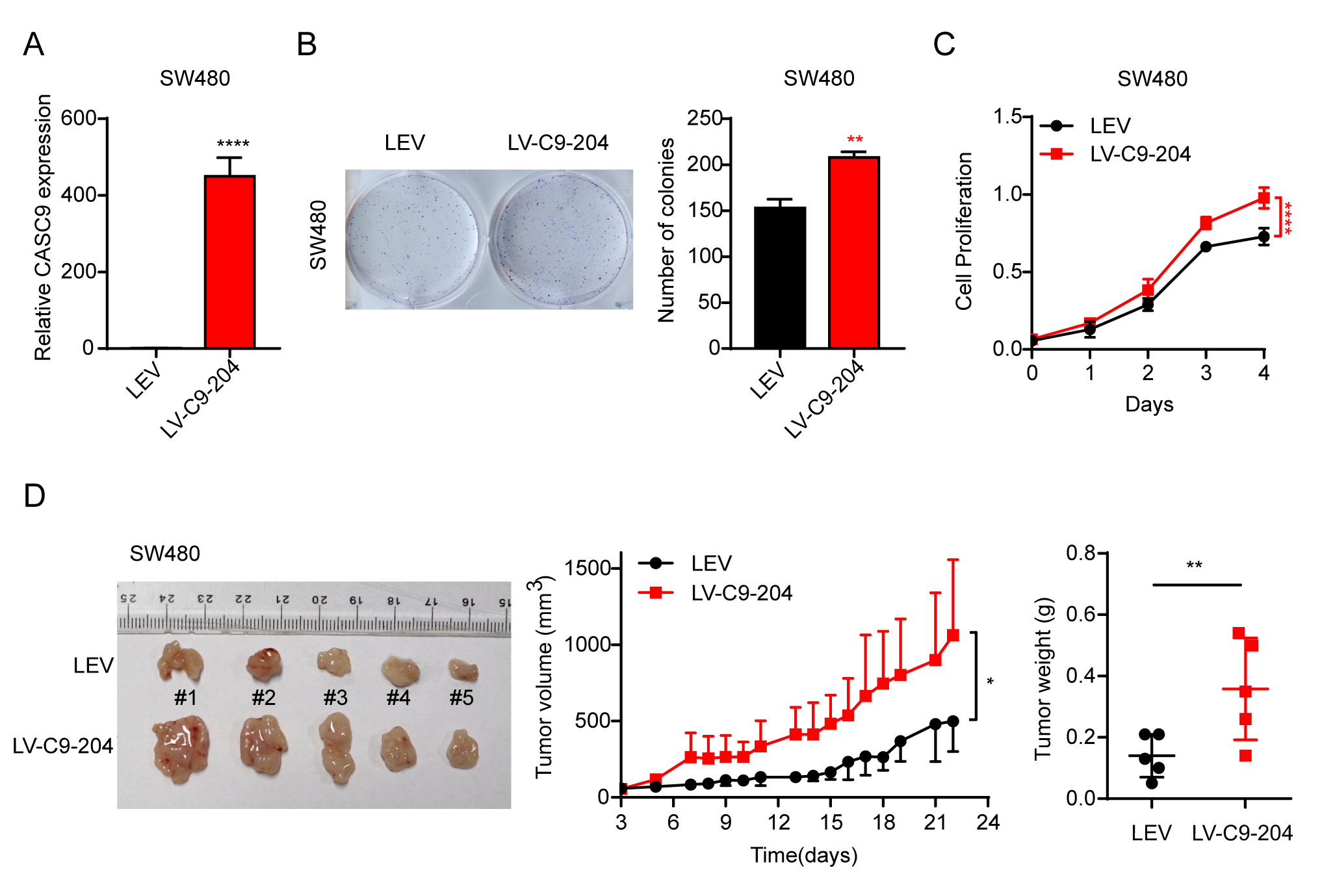

Supplement: Supplementary file 7 — Figure S6. Ectopic expression of CASC9–204 promotes proliferation of CRC cells in vitro. SW480 cells were transduced with CASC9–204-overexpressing lentivirus (LV-C9–204) or control lentivirus (LEV). (A) RT-qPCR analysis of relative CASC9 levels in LV-C9–204-SW480 cells. (B, C) Cell proliferation was determined by colony formation assay (B) and, at the indicated time points, by MTS assay (C). (D) Subcutaneous xenografts of SW480 cells transduced with LV-C9–204 or LEV (n = 5). Images of the tumors at autopsy from nude mice are presented (left), the tumor volumes were measured at the indicated time points (middle), and the average weight of the xenografted tumors was measured (right). The data are presented as mean ± s.d. **P < 0.01, ****P < 0.0001 by Student’s t-test (A, B, D) or two-way ANOVA (C, D). (TIF 2037 kb) [file 13046_2019_1263_MOESM7_ESM.tif]

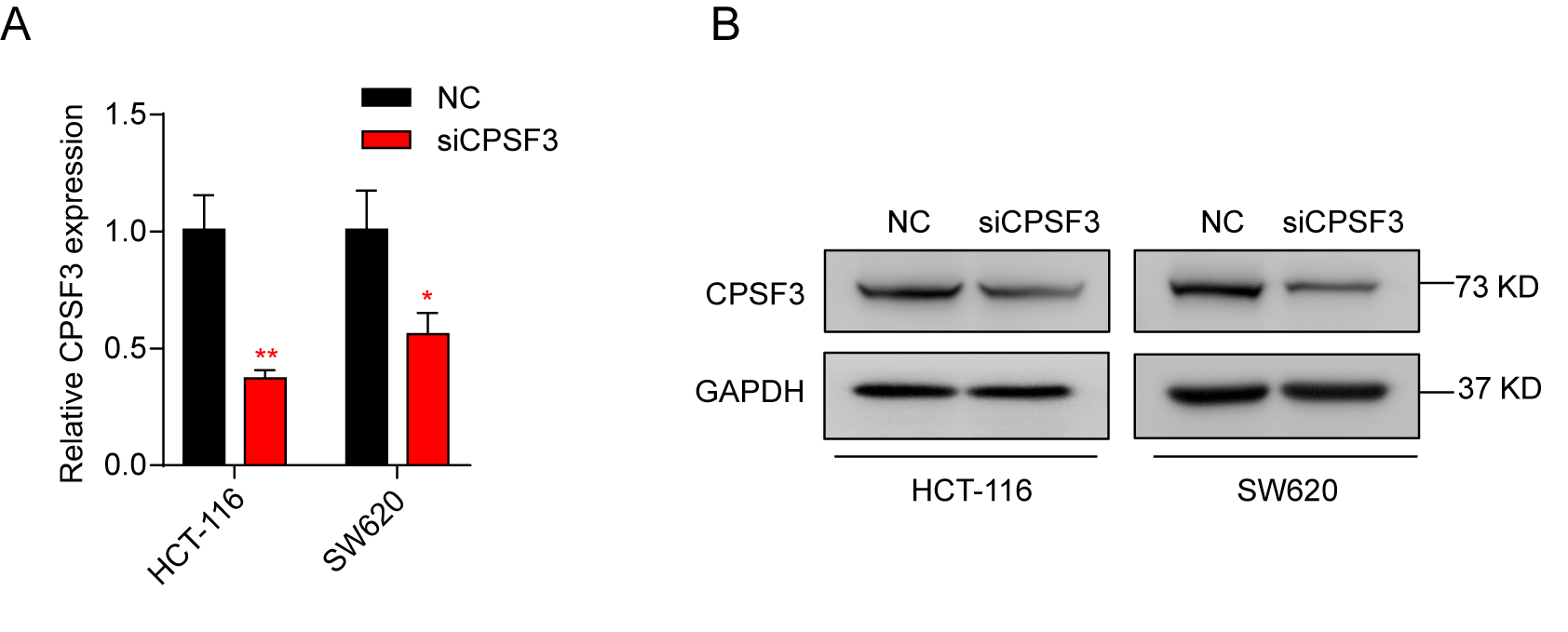

Supplement: Supplementary file 9 — Figure S7. Knockdown efficiency of CPSF3 was determined in HCT-116 and SW620 cells transfected with siRNA targeting CPSF3 (siCPSF3) or negative control (NC). (A) RT-qPCR analysis of relative CPSF3 mRNA levels. (B) Western blotting analysis of protein levels of CPSF3. The data are presented as mean ± s.d. *P < 0.05, **P < 0.01 by Student’s t-test. (TIF 537 kb) [file 13046_2019_1263_MOESM9_ESM.tif]

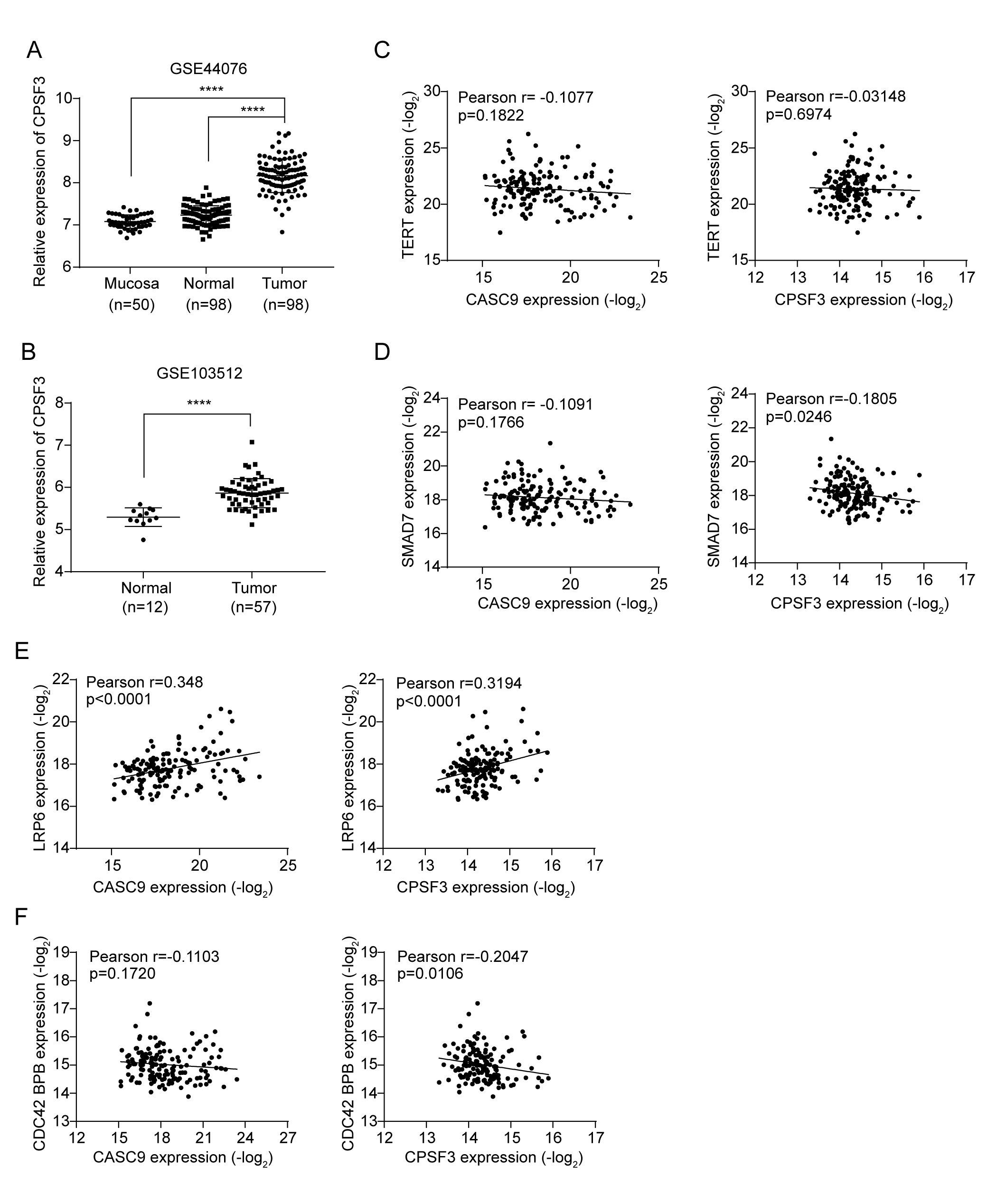

Supplement: Supplementary file 10 — Figure S8. CPSF3 was upregulated in CRC tissues. (A, B) Scatter plot showing that the expression of CPSF3 was significantly upregulated in CRC tissues compared with levels in adjacent or non-adjacent normal colon tissues in the GSE44076 (A) and GSE103512 (B) datasets. (C, D, E, F) Correlations of CASC9 and TERT (C, left), of CPSF3 and TERT (C, right), of CASC9 and SMAD7 (D, left), of CPSF3 and SMAD7 (D, right), CASC9 and LRP6 (E, left), of CPSF3 and LRP6 (E, right), and CASC9 and CDC42 BPB (F, left), of CPSF3 and CDC42 BPB (F, right), expression in 155 human CRC tissues. Expression data for CASC9, CPSF3, SMAD7, TERT, LRP6, and CDC42 BPB were downloaded from the lncRNAtor database. The data are presented as mean ± s.d. ****P < 0.0001 by Wilcoxon signed-rank test or Mann-Whitney U-test. (TIF 1526 kb) [file 13046_2019_1263_MOESM10_ESM.tif]

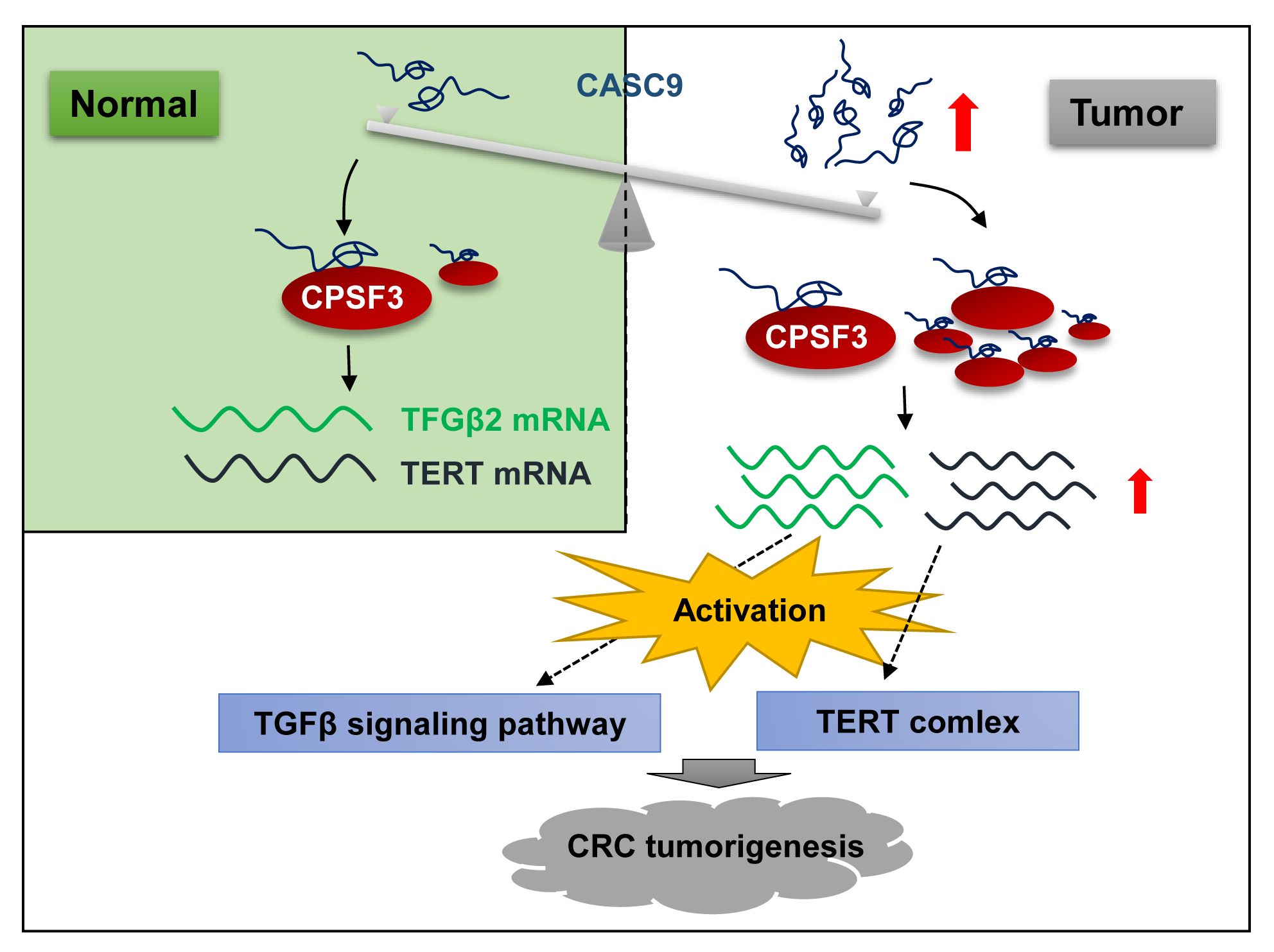

Supplement: Supplementary file 11 — Figure S9. Schematic diagram of lncRNA CASC9 interacting with CPSF3 to co-regulate genes linked to TGF-β signaling and TERT complex function and its roles in CRC tumorigenesis. (TIF 1043 kb) [file 13046_2019_1263_MOESM11_ESM.tif]
