# Supplementary material for: LncRNA CASC9 interacts with CPSF3 to regulate TGF-β signaling in colorectal cancer
Source: J Exp Clin Cancer Res. 2019 Jun 11;38:249. doi: 10.1186/s13046-019-1263-3 (PMC6560732; doi:10.1186/s13046-019-1263-3)
Supplement: Supplementary file 8 — Table S2. Gene differential expression in HCT-116/GapC9 and HCT-116/GapNC cells. (DOCX 136 kb) [file 13046_2019_1263_MOESM8_ESM.docx]

| **Additional file 8: Table S2 Gene differential expression in HCT-116/GapC9 and HCT-116/GapCON cells** | | | | | |
| --- | --- | --- | --- | --- | --- |
| **Gene name** | **Relative count (FPKM)** | | **Fold-change** | ***P* value** | **Regulation** |
|  | **GapNC** | **GapC9** | **(GapC9/GapNC)** |  |  |
| PDP1 | 4.22 | 30.96 | 7.34 | 0.000 | up |
| AADACP1 | 2.95 | 14.98 | 5.08 | 0.000 | up |
| ARPP19 | 7.76 | 40.47 | 5.21 | 0.000 | up |
| C1QL4 | 0.11 | 4.00 | 35.92 | 0.000 | up |
| AC013268.3 | 0.09 | 1.93 | 22.26 | 0.000 | up |
| AP000766.1 | 0.00 | 0.24 | inf | 0.000 | up |
| CTGF | 9.79 | 35.58 | 3.63 | 0.000 | up |
| AC024560.2 | 0.00 | 0.58 | inf | 0.000 | up |
| AC074033.1 | 0.00 | 86.30 | inf | 0.000 | up |
| RBM15B | 0.00 | 5.16 | inf | 0.000 | up |
| ORAI1 | 0.06 | 2.57 | 45.71 | 0.000 | up |
| AC078819.1 | 0.00 | 34.89 | inf | 0.000 | up |
| HASPIN | 0.00 | 6.73 | inf | 0.000 | up |
| WNK4 | 0.73 | 3.59 | 4.93 | 0.000 | up |
| AC012360.3 | 0.00 | 1.90 | inf | 0.000 | up |
| SNORA65 | 1.24 | 17.53 | 14.18 | 0.000 | up |
| LINC00294 | 0.00 | 1.73 | inf | 0.000 | up |
| C6orf62;AL031775.1 | 9.34 | 24.80 | 2.65 | 0.000 | up |
| AC008124.1 | 0.00 | 0.50 | inf | 0.000 | up |
| ZBTB5 | 0.12 | 4.43 | 36.23 | 0.000 | up |
| COL7A1 | 0.24 | 1.20 | 4.95 | 0.000 | up |
| MIS12 | 1.21 | 5.41 | 4.46 | 0.000 | up |
| ARHGEF35 | 0.23 | 6.56 | 28.32 | 0.000 | up |
| AL353796.1 | 0.00 | 1.15 | inf | 0.000 | up |
| TCEANC2 | 0.14 | 0.74 | 5.33 | 0.000 | up |
| CLDN23 | 0.00 | 1.27 | inf | 0.000 | up |
| MCM8-AS1 | 0.00 | 3.34 | inf | 0.000 | up |
| AL050341.2 | 0.00 | 1.44 | inf | 0.000 | up |
| AC245140.2 | 0.00 | 1.11 | inf | 0.000 | up |
| RIOX1 | 0.00 | 0.85 | inf | 0.000 | up |
| SMNDC1 | 7.18 | 18.11 | 2.52 | 0.000 | up |
| SP5 | 0.28 | 2.51 | 9.07 | 0.000 | up |
| KRT8P3 | 0.00 | 1.08 | inf | 0.000 | up |
| AC097639.1 | 0.00 | 0.82 | inf | 0.000 | up |
| ZNF28 | 0.98 | 3.38 | 3.44 | 0.000 | up |
| RPL7P23 | 0.00 | 1.82 | inf | 0.000 | up |
| AC112777.1 | 0.00 | 0.90 | inf | 0.000 | up |
| CBLL1 | 5.82 | 19.75 | 3.40 | 0.000 | up |
| TMEM101 | 3.57 | 10.54 | 2.95 | 0.000 | up |
| AC092368.3 | 0.00 | 0.60 | inf | 0.000 | up |
| AC079414.3 | 0.00 | 0.86 | inf | 0.000 | up |
| AC068647.2 | 1.39 | 6.47 | 4.66 | 0.000 | up |
| AL137802.2 | 0.00 | 0.76 | inf | 0.000 | up |
| AC020915.4 | 0.06 | 1.27 | 20.38 | 0.000 | up |
| REEP5 | 10.26 | 28.87 | 2.81 | 0.000 | up |
| POGZ | 4.51 | 9.80 | 2.17 | 0.000 | up |
| ZNF204P | 0.00 | 0.32 | inf | 0.000 | up |
| CD55;AL391597.1 | 7.82 | 17.60 | 2.25 | 0.000 | up |
| AL662844.4 | 0.00 | 0.24 | inf | 0.000 | up |
| LAMB3;MIR4260 | 23.26 | 46.85 | 2.01 | 0.000 | up |
| MSH4 | 0.10 | 0.71 | 7.04 | 0.000 | up |
| ZFP1 | 0.97 | 3.44 | 3.57 | 0.000 | up |
| HSD17B1 | 0.17 | 0.84 | 5.05 | 0.000 | up |
| GPATCH2L;AC016526.2 | 0.76 | 2.87 | 3.77 | 0.000 | up |
| KMT2E | 5.42 | 10.90 | 2.01 | 0.000 | up |
| TSC22D2 | 0.61 | 2.99 | 4.92 | 0.000 | up |
| KLHL11 | 0.31 | 8.40 | 27.16 | 0.000 | up |
| GATAD1;AC007566.1 | 1.51 | 4.30 | 2.84 | 0.000 | up |
| LINC00973 | 1.76 | 6.46 | 3.66 | 0.000 | up |
| IL10RB-AS1 | 0.05 | 1.10 | 22.68 | 0.000 | up |
| ZNF449 | 0.52 | 1.92 | 3.68 | 0.000 | up |
| SLC36A1 | 1.01 | 2.70 | 2.67 | 0.000 | up |
| N6AMT1 | 0.65 | 2.06 | 3.16 | 0.000 | up |
| CAMK2N1 | 3.48 | 8.87 | 2.55 | 0.000 | up |
| TP53INP1 | 3.63 | 8.04 | 2.21 | 0.000 | up |
| OR2A7 | 0.21 | 1.80 | 8.47 | 0.000 | up |
| TCEA1P2 | 0.53 | 21.47 | 40.88 | 0.000 | up |
| TNFAIP3 | 2.22 | 5.22 | 2.35 | 0.000 | up |
| GABARAPL1 | 4.74 | 10.01 | 2.11 | 0.000 | up |
| ZNF518B | 1.80 | 6.16 | 3.42 | 0.000 | up |
| COL11A2 | 0.75 | 2.03 | 2.68 | 0.000 | up |
| CSTF3 | 5.73 | 11.90 | 2.08 | 0.001 | up |
| HCN2 | 1.10 | 4.21 | 3.83 | 0.001 | up |
| THSD1 | 0.27 | 1.03 | 3.89 | 0.001 | up |
| GABPA | 6.51 | 13.40 | 2.06 | 0.001 | up |
| MPZL3 | 2.50 | 5.80 | 2.32 | 0.001 | up |
| SLFN5 | 0.97 | 2.27 | 2.34 | 0.001 | up |
| MGAT1 | 0.94 | 3.84 | 4.07 | 0.001 | up |
| TMEM110-MUSTN1 | 0.01 | 0.16 | 24.85 | 0.001 | up |
| AMD1 | 8.65 | 18.77 | 2.17 | 0.001 | up |
| ZNF468 | 4.54 | 9.41 | 2.07 | 0.001 | up |
| ATF3 | 6.03 | 12.26 | 2.03 | 0.001 | up |
| ZNF503;AC010997.3 | 7.10 | 21.32 | 3.00 | 0.001 | up |
| ZNF559 | 0.35 | 1.11 | 3.20 | 0.001 | up |
| GPD1L | 2.52 | 6.34 | 2.51 | 0.001 | up |
| UBA5 | 2.26 | 4.95 | 2.19 | 0.001 | up |
| ZNF24 | 11.84 | 24.63 | 2.08 | 0.001 | up |
| H2AFJ | 0.11 | 0.66 | 6.22 | 0.001 | up |
| HNRNPA3P6;MIR4444-2 | 1.19 | 34.15 | 28.75 | 0.001 | up |
| AADAC | 1.44 | 4.77 | 3.32 | 0.001 | up |
| THEM4 | 1.91 | 4.70 | 2.47 | 0.001 | up |
| ZNF225 | 0.45 | 1.48 | 3.31 | 0.001 | up |
| CCDC62 | 0.49 | 1.46 | 3.02 | 0.001 | up |
| TRAPPC6B | 2.34 | 6.37 | 2.72 | 0.001 | up |
| EPC1 | 1.74 | 3.56 | 2.04 | 0.001 | up |
| XIAP | 1.95 | 4.28 | 2.19 | 0.001 | up |
| AC108174.1 | 0.20 | 2.28 | 11.18 | 0.001 | up |
| TXLNA | 7.54 | 16.63 | 2.21 | 0.001 | up |
| FCER1G | 0.18 | 0.99 | 5.44 | 0.001 | up |
| PPIL1 | 8.85 | 23.18 | 2.62 | 0.001 | up |
| FAM111B | 0.35 | 2.09 | 5.98 | 0.001 | up |
| RAB30 | 0.12 | 0.65 | 5.41 | 0.001 | up |
| LTA | 0.13 | 0.73 | 5.67 | 0.002 | up |
| AC003002.3 | 0.12 | 1.85 | 15.93 | 0.002 | up |
| ZFP36L1 | 18.38 | 40.92 | 2.23 | 0.002 | up |
| EDN1 | 1.06 | 3.03 | 2.87 | 0.002 | up |
| DANCR | 4.84 | 10.41 | 2.15 | 0.002 | up |
| RASIP1 | 0.16 | 0.58 | 3.66 | 0.002 | up |
| WNT8B | 0.14 | 0.61 | 4.40 | 0.002 | up |
| GPD1 | 0.21 | 0.77 | 3.75 | 0.002 | up |
| AGAP6 | 2.42 | 5.58 | 2.31 | 0.002 | up |
| VPS37B;AC027290.2 | 1.14 | 2.44 | 2.15 | 0.002 | up |
| ZNF670 | 0.37 | 1.82 | 4.91 | 0.002 | up |
| LINC00324 | 0.22 | 0.93 | 4.18 | 0.002 | up |
| EIF4BP7 | 1.13 | 4.68 | 4.14 | 0.002 | up |
| TP53INP2 | 2.79 | 5.76 | 2.06 | 0.002 | up |
| KCTD2 | 2.84 | 8.34 | 2.93 | 0.002 | up |
| TSKU | 0.97 | 3.32 | 3.44 | 0.003 | up |
| SNORA66 | 1.39 | 6.69 | 4.82 | 0.003 | up |
| BTG1 | 2.04 | 4.20 | 2.06 | 0.003 | up |
| IMPAD1 | 3.37 | 10.00 | 2.97 | 0.003 | up |
| ZBTB41 | 3.01 | 6.53 | 2.17 | 0.003 | up |
| SBSN | 0.16 | 0.64 | 3.97 | 0.003 | up |
| SEMA3A | 1.51 | 3.05 | 2.02 | 0.003 | up |
| TMCC3 | 0.56 | 1.79 | 3.19 | 0.004 | up |
| TIGD2 | 0.25 | 1.42 | 5.69 | 0.004 | up |
| LINC00847 | 0.62 | 1.83 | 2.97 | 0.004 | up |
| SSR3 | 14.25 | 34.11 | 2.39 | 0.004 | up |
| SKIL | 0.72 | 1.58 | 2.20 | 0.005 | up |
| MIR22HG | 1.32 | 3.44 | 2.61 | 0.005 | up |
| GCNA | 0.30 | 0.89 | 2.95 | 0.005 | up |
| PEX2 | 1.80 | 4.69 | 2.60 | 0.005 | up |
| EXTL2 | 1.64 | 3.63 | 2.22 | 0.005 | up |
| ZMAT3 | 2.19 | 4.75 | 2.16 | 0.005 | up |
| ANG | 0.86 | 2.49 | 2.90 | 0.005 | up |
| TROVE2 | 2.01 | 4.06 | 2.02 | 0.005 | up |
| SMG1P7 | 0.35 | 0.97 | 2.81 | 0.005 | up |
| ZNF791 | 0.45 | 1.25 | 2.76 | 0.005 | up |
| ZNF233 | 0.09 | 0.38 | 4.02 | 0.006 | up |
| CPEB4 | 0.56 | 1.44 | 2.56 | 0.006 | up |
| OTUD3 | 5.84 | 12.65 | 2.17 | 0.006 | up |
| AL161431.1 | 14.79 | 39.40 | 2.66 | 0.006 | up |
| ZNF699 | 0.33 | 1.25 | 3.82 | 0.006 | up |
| SLC35B2 | 5.74 | 15.00 | 2.61 | 0.006 | up |
| LRRN4CL | 0.29 | 0.94 | 3.20 | 0.006 | up |
| KLHL23;PHOSPHO2 | 5.09 | 10.26 | 2.01 | 0.007 | up |
| FOSB | 0.17 | 0.55 | 3.29 | 0.007 | up |
| BCL6 | 1.43 | 2.89 | 2.02 | 0.007 | up |
| TCTE1 | 0.11 | 0.40 | 3.73 | 0.007 | up |
| RAB36 | 0.90 | 2.05 | 2.29 | 0.007 | up |
| ZNF808 | 0.25 | 0.71 | 2.89 | 0.007 | up |
| INTS6-AS1;RPS4XP16 | 0.08 | 0.26 | 3.07 | 0.007 | up |
| AC097478.1 | 0.66 | 1.53 | 2.30 | 0.008 | up |
| SP9 | 0.13 | 0.45 | 3.58 | 0.008 | up |
| ZNF547 | 0.08 | 0.27 | 3.65 | 0.008 | up |
| BNIPL | 0.22 | 0.67 | 3.09 | 0.008 | up |
| CCPG1 | 0.92 | 1.85 | 2.01 | 0.008 | up |
| ENTPD1-AS1 | 0.30 | 0.66 | 2.24 | 0.008 | up |
| FAM72C | 0.74 | 2.68 | 3.63 | 0.008 | up |
| IFFO1;AC006064.4 | 0.19 | 0.88 | 4.71 | 0.009 | up |
| ACSBG1 | 0.06 | 0.24 | 3.78 | 0.009 | up |
| NUP50-AS1 | 0.49 | 1.76 | 3.59 | 0.009 | up |
| ZNF146 | 20.22 | 42.18 | 2.09 | 0.009 | up |
| AC008393.1 | 0.18 | 0.63 | 3.51 | 0.010 | up |
| MUC12 | 0.03 | 0.11 | 3.49 | 0.010 | up |
| SMAD7 | 0.96 | 2.15 | 2.24 | 0.010 | up |
| DRAM1 | 4.18 | 9.39 | 2.25 | 0.010 | up |
| UMAD1;GLCCI1;AC007161.3 | 0.52 | 1.08 | 2.06 | 0.010 | up |
| PICART1 | 0.37 | 1.07 | 2.90 | 0.010 | up |
| FAM136A | 22.41 | 53.25 | 2.38 | 0.011 | up |
| RUNDC3A-AS1 | 0.16 | 0.46 | 2.84 | 0.012 | up |
| HOXC5 | 1.32 | 3.55 | 2.68 | 0.012 | up |
| MIS18A | 7.77 | 16.08 | 2.07 | 0.012 | up |
| ZNF155 | 0.10 | 0.35 | 3.39 | 0.012 | up |
| AKIRIN2 | 4.70 | 10.95 | 2.33 | 0.012 | up |
| WHAMMP3 | 0.29 | 0.68 | 2.38 | 0.013 | up |
| PCBD2 | 0.48 | 1.20 | 2.48 | 0.013 | up |
| ZNF177 | 0.05 | 0.50 | 10.95 | 0.013 | up |
| WHAMMP2 | 0.17 | 0.51 | 2.95 | 0.013 | up |
| CFAP53 | 0.77 | 1.88 | 2.43 | 0.013 | up |
| LINC02535 | 0.98 | 2.30 | 2.35 | 0.014 | up |
| LYRM9 | 0.07 | 0.25 | 3.44 | 0.014 | up |
| ZNF641 | 0.16 | 0.46 | 2.84 | 0.014 | up |
| HOXA11-AS | 0.13 | 1.02 | 7.77 | 0.014 | up |
| AC135506.1 | 0.23 | 0.73 | 3.18 | 0.014 | up |
| C4orf46 | 2.80 | 6.61 | 2.36 | 0.015 | up |
| HS6ST2 | 1.29 | 3.35 | 2.59 | 0.015 | up |
| AC092683.1 | 0.78 | 1.63 | 2.08 | 0.016 | up |
| AC110079.1 | 0.99 | 2.78 | 2.81 | 0.016 | up |
| NABP1 | 0.72 | 1.71 | 2.37 | 0.016 | up |
| ZSCAN31 | 0.53 | 1.27 | 2.39 | 0.016 | up |
| HOXC-AS1 | 0.37 | 1.35 | 3.65 | 0.017 | up |
| RUNDC3A | 0.07 | 0.24 | 3.20 | 0.017 | up |
| LRRC37A3 | 0.48 | 1.01 | 2.09 | 0.018 | up |
| SPON1 | 0.10 | 0.29 | 2.88 | 0.018 | up |
| NUDT19 | 4.77 | 11.20 | 2.35 | 0.019 | up |
| ZNF564 | 0.22 | 0.84 | 3.86 | 0.019 | up |
| TBR1;AC009487.3 | 0.02 | 0.07 | 3.88 | 0.020 | up |
| MED18 | 1.65 | 4.21 | 2.54 | 0.020 | up |
| BTN3A1 | 0.31 | 0.70 | 2.26 | 0.022 | up |
| NLRC3 | 0.12 | 0.32 | 2.67 | 0.022 | up |
| SUMO3 | 11.59 | 24.99 | 2.16 | 0.022 | up |
| CHAC1 | 0.71 | 1.95 | 2.75 | 0.022 | up |
| SNX18 | 1.44 | 4.53 | 3.14 | 0.022 | up |
| MYOM1 | 0.09 | 0.24 | 2.74 | 0.022 | up |
| ZNF780A | 0.20 | 0.49 | 2.44 | 0.022 | up |
| ZNF784 | 0.21 | 0.86 | 4.14 | 0.023 | up |
| ZNF816 | 0.34 | 0.86 | 2.50 | 0.024 | up |
| PTENP1 | 0.12 | 0.37 | 3.10 | 0.024 | up |
| P2RX2 | 0.17 | 0.48 | 2.87 | 0.025 | up |
| AC104986.2 | 2.04 | 5.80 | 2.85 | 0.026 | up |
| HOXC9 | 1.44 | 3.54 | 2.46 | 0.026 | up |
| AC093323.1 | 2.86 | 6.41 | 2.25 | 0.026 | up |
| GALR2 | 0.24 | 0.74 | 3.05 | 0.026 | up |
| TRIM62 | 0.42 | 1.19 | 2.84 | 0.027 | up |
| RBM43 | 0.15 | 0.59 | 3.88 | 0.027 | up |
| NAT16 | 0.18 | 0.48 | 2.64 | 0.028 | up |
| PPP1R26-AS1 | 0.58 | 1.21 | 2.10 | 0.028 | up |
| SPRY4 | 3.19 | 6.68 | 2.09 | 0.029 | up |
| MORN4 | 1.16 | 2.57 | 2.22 | 0.029 | up |
| GPAT2P2 | 0.30 | 0.90 | 3.00 | 0.029 | up |
| GTF2IRD2B | 0.39 | 0.82 | 2.13 | 0.029 | up |
| ZNF611 | 0.36 | 0.77 | 2.14 | 0.030 | up |
| CD27-AS1 | 0.12 | 0.35 | 2.88 | 0.031 | up |
| FAM214A | 0.32 | 0.66 | 2.08 | 0.033 | up |
| CAND2 | 0.17 | 0.41 | 2.39 | 0.033 | up |
| PNLDC1 | 0.11 | 0.32 | 2.94 | 0.034 | up |
| ANKRD29 | 0.44 | 0.89 | 2.02 | 0.034 | up |
| NLGN3 | 0.65 | 1.33 | 2.03 | 0.035 | up |
| RAG1 | 0.12 | 0.30 | 2.37 | 0.035 | up |
| HAP1 | 0.40 | 0.87 | 2.15 | 0.036 | up |
| EGR3 | 0.37 | 0.77 | 2.11 | 0.036 | up |
| CKMT2-AS1 | 1.10 | 2.20 | 2.00 | 0.036 | up |
| THAP10 | 0.70 | 1.45 | 2.09 | 0.037 | up |
| CLHC1 | 0.40 | 0.87 | 2.20 | 0.037 | up |
| ACTBP13 | 0.08 | 0.22 | 2.65 | 0.037 | up |
| MIATNB | 0.23 | 0.57 | 2.49 | 0.037 | up |
| AL354920.1 | 0.09 | 0.60 | 6.68 | 0.039 | up |
| GPRASP1 | 0.09 | 0.31 | 3.51 | 0.039 | up |
| LINC00672 | 0.21 | 0.50 | 2.37 | 0.039 | up |
| ZNF227 | 1.82 | 3.64 | 2.00 | 0.040 | up |
| BZW1P2 | 23.75 | 70.03 | 2.95 | 0.042 | up |
| HMGN5 | 0.57 | 1.27 | 2.23 | 0.043 | up |
| TMEM242 | 1.77 | 3.65 | 2.07 | 0.044 | up |
| FOXP4-AS1 | 0.41 | 1.06 | 2.61 | 0.047 | up |
| EIF4EBP2 | 3.93 | 13.73 | 3.50 | 0.048 | up |
| FOXA1 | 0.12 | 0.30 | 2.47 | 0.049 | up |
| ADHFE1;RRS1;C8orf46 | 0.28 | 0.79 | 2.88 | 0.049 | up |
| POC1B-GALNT4 | 0.29 | 1.20 | 4.09 | 0.049 | up |
| CCDC85B | 31.09 | 0.00 | -inf | 0.000 | down |
| NSMCE3 | 2.96 | 0.00 | -inf | 0.000 | down |
| TIGD5 | 2.37 | 0.00 | -inf | 0.000 | down |
| ERCC6L | 6.86 | 0.11 | 0.02 | 0.000 | down |
| NPIPB4 | 3.62 | 0.00 | -inf | 0.000 | down |
| FOXD1 | 2.37 | 0.01 | 0.00 | 0.000 | down |
| AL121603.2 | 0.50 | 0.00 | -inf | 0.000 | down |
| ASAP1 | 14.97 | 4.70 | 0.31 | 0.000 | down |
| NPLOC4 | 21.06 | 6.04 | 0.29 | 0.000 | down |
| CCDC117 | 18.35 | 4.82 | 0.26 | 0.000 | down |
| TMEM181 | 13.75 | 3.43 | 0.25 | 0.000 | down |
| MSL2 | 5.75 | 0.05 | 0.01 | 0.000 | down |
| MT1E | 89.12 | 25.80 | 0.29 | 0.000 | down |
| TRRAP;AC004893.2 | 12.28 | 4.53 | 0.37 | 0.000 | down |
| IER2 | 48.70 | 3.13 | 0.06 | 0.000 | down |
| RNF139 | 11.66 | 0.08 | 0.01 | 0.000 | down |
| AC083843.2 | 0.66 | 0.00 | -inf | 0.000 | down |
| SMIM12 | 2.42 | 0.22 | 0.09 | 0.000 | down |
| AC099336.2 | 11.21 | 0.00 | -inf | 0.000 | down |
| MLXIP | 14.42 | 5.56 | 0.39 | 0.000 | down |
| AC010969.2 | 0.82 | 0.00 | -inf | 0.000 | down |
| RCN1P2 | 2.78 | 0.00 | -inf | 0.000 | down |
| TEAD1 | 9.19 | 3.06 | 0.33 | 0.000 | down |
| MEMO1P1 | 2.77 | 0.00 | -inf | 0.000 | down |
| SEPT10 | 17.39 | 5.57 | 0.32 | 0.000 | down |
| RPL7AP66 | 2.90 | 0.00 | -inf | 0.000 | down |
| ZBED2 | 26.78 | 0.97 | 0.04 | 0.000 | down |
| ADD1 | 9.98 | 4.08 | 0.41 | 0.000 | down |
| SPATS2 | 8.80 | 2.97 | 0.34 | 0.000 | down |
| ZNF322P1 | 1.01 | 0.00 | -inf | 0.000 | down |
| UCKL1-AS1 | 0.34 | 0.00 | -inf | 0.000 | down |
| CDC42BPB | 18.33 | 7.65 | 0.42 | 0.000 | down |
| LINC01311 | 0.72 | 0.00 | -inf | 0.000 | down |
| PLK4 | 9.04 | 2.82 | 0.31 | 0.000 | down |
| PSMD2 | 84.96 | 39.02 | 0.46 | 0.000 | down |
| ZNF674-AS1 | 1.93 | 0.17 | 0.09 | 0.000 | down |
| KIAA0100;SPAG5;AC005726.2;SGK494;ALDOC | 17.97 | 8.27 | 0.46 | 0.000 | down |
| AC046176.1 | 0.71 | 0.00 | -inf | 0.000 | down |
| SNRPEP4 | 2.98 | 0.00 | -inf | 0.000 | down |
| N4BP2L2-IT2 | 0.18 | 0.00 | -inf | 0.000 | down |
| CDCP1 | 69.09 | 32.26 | 0.47 | 0.000 | down |
| AC068724.1 | 0.96 | 0.00 | -inf | 0.000 | down |
| HELLS;AL138759.1 | 7.18 | 2.68 | 0.37 | 0.000 | down |
| HECTD4 | 1.84 | 0.60 | 0.33 | 0.000 | down |
| FAM157A | 1.59 | 0.30 | 0.19 | 0.000 | down |
| NCOR2 | 10.16 | 4.20 | 0.41 | 0.000 | down |
| WNK1 | 14.86 | 6.65 | 0.45 | 0.000 | down |
| MSI2 | 6.20 | 2.68 | 0.43 | 0.000 | down |
| ABCC1 | 28.72 | 12.40 | 0.43 | 0.000 | down |
| ITGA6 | 35.52 | 16.10 | 0.45 | 0.000 | down |
| AL035461.2 | 1.28 | 0.00 | -inf | 0.000 | down |
| ARHGAP35 | 9.88 | 4.13 | 0.42 | 0.000 | down |
| ARMC6 | 15.42 | 5.18 | 0.34 | 0.000 | down |
| TBK1 | 9.27 | 3.14 | 0.34 | 0.000 | down |
| RBAK;RNF216P1;RBAK-RBAKDN;RBAKDN | 3.59 | 1.17 | 0.32 | 0.000 | down |
| A4GALT | 3.05 | 0.50 | 0.16 | 0.000 | down |
| PIAS2 | 1.81 | 0.57 | 0.31 | 0.000 | down |
| H2AFY | 10.48 | 4.53 | 0.43 | 0.000 | down |
| PCSK9 | 9.19 | 3.00 | 0.33 | 0.000 | down |
| LPCAT1 | 13.00 | 5.01 | 0.39 | 0.000 | down |
| TRIM8 | 17.30 | 6.28 | 0.36 | 0.000 | down |
| RPTOR | 8.92 | 3.53 | 0.40 | 0.000 | down |
| FBXO36 | 1.07 | 0.19 | 0.18 | 0.000 | down |
| IP6K1 | 12.27 | 4.48 | 0.36 | 0.000 | down |
| SEMA6B | 10.74 | 3.93 | 0.37 | 0.000 | down |
| CHCHD4 | 13.20 | 4.23 | 0.32 | 0.000 | down |
| RAPGEF1 | 12.38 | 5.00 | 0.40 | 0.000 | down |
| PCNX1 | 3.53 | 1.35 | 0.38 | 0.000 | down |
| GGA2 | 11.69 | 4.92 | 0.42 | 0.000 | down |
| HMGA2 | 7.85 | 3.45 | 0.44 | 0.000 | down |
| AC016026.1 | 0.39 | 0.02 | 0.04 | 0.000 | down |
| ZNF510 | 1.04 | 0.18 | 0.17 | 0.000 | down |
| FAM120A | 31.03 | 14.39 | 0.46 | 0.000 | down |
| SPTBN1 | 13.64 | 6.52 | 0.48 | 0.000 | down |
| INPP4B | 2.53 | 0.83 | 0.33 | 0.000 | down |
| NAP1L4 | 25.56 | 11.65 | 0.46 | 0.000 | down |
| VPS28;TONSL | 14.22 | 6.19 | 0.44 | 0.000 | down |
| CICP14 | 3.28 | 0.79 | 0.24 | 0.000 | down |
| MAP1B | 2.78 | 0.97 | 0.35 | 0.000 | down |
| CUL3 | 10.16 | 4.52 | 0.44 | 0.000 | down |
| NAA15 | 16.76 | 7.53 | 0.45 | 0.000 | down |
| TNFAIP2 | 22.09 | 9.78 | 0.44 | 0.000 | down |
| VKORC1L1 | 12.78 | 2.30 | 0.18 | 0.000 | down |
| CACNA1H | 2.59 | 0.88 | 0.34 | 0.000 | down |
| EHBP1L1 | 16.97 | 7.80 | 0.46 | 0.000 | down |
| HIPK2 | 7.92 | 3.46 | 0.44 | 0.000 | down |
| GRINA | 39.73 | 17.68 | 0.44 | 0.000 | down |
| ENOSF1 | 3.78 | 1.45 | 0.38 | 0.000 | down |
| VAV2 | 7.66 | 2.84 | 0.37 | 0.000 | down |
| NACC1 | 31.36 | 11.14 | 0.36 | 0.000 | down |
| ADGRG6 | 15.40 | 6.80 | 0.44 | 0.000 | down |
| PHRF1 | 22.85 | 10.61 | 0.46 | 0.000 | down |
| SEL1L3 | 4.79 | 1.84 | 0.39 | 0.000 | down |
| PTPRK | 6.11 | 2.48 | 0.41 | 0.000 | down |
| CDC42 | 79.55 | 39.03 | 0.49 | 0.000 | down |
| PPIP5K1 | 3.47 | 1.29 | 0.37 | 0.000 | down |
| LRP6 | 4.66 | 1.88 | 0.40 | 0.000 | down |
| PLXNA1 | 11.62 | 5.38 | 0.46 | 0.000 | down |
| REV3L | 3.57 | 1.39 | 0.39 | 0.000 | down |
| ROR2 | 3.04 | 0.96 | 0.32 | 0.000 | down |
| MYBBP1A | 29.22 | 13.29 | 0.45 | 0.000 | down |
| HACD2 | 14.92 | 4.40 | 0.30 | 0.000 | down |
| NACC2 | 12.78 | 5.78 | 0.45 | 0.000 | down |
| PACS1 | 4.37 | 1.73 | 0.40 | 0.000 | down |
| IFRD2;NAT6;HYAL3 | 13.37 | 5.97 | 0.45 | 0.000 | down |
| COL12A1 | 12.58 | 6.22 | 0.49 | 0.000 | down |
| ABR | 4.81 | 2.04 | 0.42 | 0.000 | down |
| DAZAP1 | 16.34 | 8.11 | 0.50 | 0.000 | down |
| TCF7 | 2.06 | 0.68 | 0.33 | 0.000 | down |
| PLEKHB2 | 25.44 | 11.85 | 0.47 | 0.000 | down |
| FOXK1 | 12.91 | 6.21 | 0.48 | 0.000 | down |
| TINF2 | 6.99 | 2.46 | 0.35 | 0.000 | down |
| MTMR9 | 4.88 | 1.48 | 0.30 | 0.000 | down |
| MICAL3 | 2.77 | 1.21 | 0.44 | 0.000 | down |
| MMGT1 | 10.02 | 2.19 | 0.22 | 0.000 | down |
| ANKS1A | 5.92 | 2.46 | 0.42 | 0.000 | down |
| B3GALNT2 | 4.37 | 1.67 | 0.38 | 0.001 | down |
| DPYSL5 | 2.00 | 0.63 | 0.31 | 0.001 | down |
| DENND5B | 5.82 | 2.60 | 0.45 | 0.001 | down |
| CABIN1 | 6.57 | 2.90 | 0.44 | 0.001 | down |
| MAFG;SIRT7 | 9.42 | 4.00 | 0.42 | 0.001 | down |
| MGAT5 | 7.93 | 3.52 | 0.44 | 0.001 | down |
| FAM83H | 11.96 | 5.17 | 0.43 | 0.001 | down |
| COX14;AC074032.1 | 3.63 | 0.51 | 0.14 | 0.001 | down |
| CNOT9 | 19.42 | 8.85 | 0.46 | 0.001 | down |
| KIF4A | 17.63 | 8.27 | 0.47 | 0.001 | down |
| SPRED1 | 5.27 | 2.17 | 0.41 | 0.001 | down |
| PRKAA1 | 11.40 | 4.85 | 0.43 | 0.001 | down |
| UBN2 | 1.92 | 0.62 | 0.32 | 0.001 | down |
| SKI | 11.74 | 4.48 | 0.38 | 0.001 | down |
| CAPN15 | 9.18 | 4.07 | 0.44 | 0.001 | down |
| PIKFYVE | 4.18 | 1.81 | 0.43 | 0.001 | down |
| PLCB3 | 32.44 | 16.12 | 0.50 | 0.001 | down |
| SIPA1L3 | 5.05 | 2.18 | 0.43 | 0.001 | down |
| MPHOSPH6 | 21.42 | 9.07 | 0.42 | 0.001 | down |
| TAF3 | 5.40 | 2.14 | 0.40 | 0.001 | down |
| NPAS2 | 0.88 | 0.29 | 0.32 | 0.001 | down |
| OSBPL3 | 8.21 | 3.66 | 0.45 | 0.001 | down |
| SH3D19 | 3.90 | 1.65 | 0.42 | 0.001 | down |
| WDR90 | 5.04 | 2.11 | 0.42 | 0.001 | down |
| MSRB3 | 8.50 | 3.74 | 0.44 | 0.001 | down |
| PI4KAP1 | 1.77 | 0.51 | 0.29 | 0.001 | down |
| EPB41L1 | 4.79 | 2.08 | 0.43 | 0.001 | down |
| DHPS | 12.83 | 5.45 | 0.42 | 0.001 | down |
| ABCC10 | 3.17 | 1.25 | 0.39 | 0.001 | down |
| INPP5F | 7.16 | 2.99 | 0.42 | 0.001 | down |
| GINS1 | 10.00 | 3.52 | 0.35 | 0.001 | down |
| KDM6A | 3.07 | 1.21 | 0.39 | 0.001 | down |
| HSPBP1 | 22.10 | 9.45 | 0.43 | 0.001 | down |
| KMT2C | 3.17 | 1.46 | 0.46 | 0.001 | down |
| TERT | 1.32 | 0.37 | 0.28 | 0.001 | down |
| SPATA2L | 8.28 | 3.26 | 0.39 | 0.001 | down |
| SCARB1 | 11.11 | 5.39 | 0.48 | 0.001 | down |
| TANC1 | 2.60 | 1.03 | 0.40 | 0.001 | down |
| HPSE | 5.42 | 2.20 | 0.41 | 0.001 | down |
| FRMD5 | 9.43 | 4.12 | 0.44 | 0.001 | down |
| MEGF6 | 1.93 | 0.65 | 0.34 | 0.001 | down |
| ZBTB37 | 1.12 | 0.23 | 0.21 | 0.001 | down |
| BCAS4 | 2.24 | 0.68 | 0.30 | 0.001 | down |
| LSS | 11.82 | 5.75 | 0.49 | 0.001 | down |
| SAV1 | 2.82 | 0.60 | 0.21 | 0.001 | down |
| AL732372.3 | 5.30 | 1.51 | 0.28 | 0.001 | down |
| C20orf194 | 1.24 | 0.43 | 0.34 | 0.001 | down |
| TULP4 | 3.21 | 1.33 | 0.41 | 0.001 | down |
| ENTPD6 | 11.11 | 5.16 | 0.46 | 0.001 | down |
| IBA57 | 1.53 | 0.37 | 0.24 | 0.001 | down |
| COQ8A | 4.09 | 1.78 | 0.44 | 0.002 | down |
| LRRC59 | 107.10 | 48.60 | 0.45 | 0.002 | down |
| CATSPER2;AC011330.1 | 1.00 | 0.35 | 0.35 | 0.002 | down |
| TNS3 | 3.87 | 1.67 | 0.43 | 0.002 | down |
| CARS2 | 3.64 | 1.65 | 0.45 | 0.002 | down |
| CEP192 | 3.53 | 1.55 | 0.44 | 0.002 | down |
| ANTXR1 | 4.89 | 2.21 | 0.45 | 0.002 | down |
| ZNF675 | 0.32 | 0.06 | 0.20 | 0.002 | down |
| AUH | 4.97 | 1.73 | 0.35 | 0.002 | down |
| HPRT1 | 31.78 | 13.74 | 0.43 | 0.002 | down |
| TSC2 | 6.07 | 2.92 | 0.48 | 0.002 | down |
| PIK3C3 | 1.55 | 0.64 | 0.41 | 0.002 | down |
| SLC12A7 | 10.30 | 4.79 | 0.46 | 0.002 | down |
| WWC2 | 4.93 | 2.19 | 0.44 | 0.002 | down |
| CDH24 | 8.18 | 3.63 | 0.44 | 0.002 | down |
| CHSY1 | 9.93 | 4.73 | 0.48 | 0.002 | down |
| CLEC16A | 3.27 | 1.51 | 0.46 | 0.002 | down |
| TBC1D30 | 4.57 | 2.13 | 0.47 | 0.002 | down |
| SMG6 | 3.72 | 1.65 | 0.44 | 0.002 | down |
| ENAH | 2.82 | 1.32 | 0.47 | 0.002 | down |
| DGKZ | 7.91 | 3.95 | 0.50 | 0.002 | down |
| CENPI | 3.48 | 1.34 | 0.39 | 0.002 | down |
| PAAF1 | 1.45 | 0.54 | 0.37 | 0.002 | down |
| HPCAL1 | 8.71 | 4.04 | 0.46 | 0.002 | down |
| SLC38A10 | 8.76 | 4.12 | 0.47 | 0.002 | down |
| ADD3 | 3.90 | 1.61 | 0.41 | 0.002 | down |
| ZNF746 | 3.45 | 1.34 | 0.39 | 0.002 | down |
| CORO1A | 1.83 | 0.64 | 0.35 | 0.002 | down |
| SAR1A | 10.66 | 4.86 | 0.46 | 0.003 | down |
| PRR3 | 5.02 | 1.58 | 0.32 | 0.003 | down |
| CISD3 | 15.24 | 5.96 | 0.39 | 0.003 | down |
| GREB1 | 0.71 | 0.24 | 0.35 | 0.003 | down |
| RGPD6 | 2.39 | 1.08 | 0.45 | 0.003 | down |
| WNK2 | 2.19 | 0.95 | 0.44 | 0.003 | down |
| TMEM168 | 2.85 | 1.20 | 0.42 | 0.003 | down |
| KCNAB2 | 2.32 | 0.94 | 0.41 | 0.003 | down |
| CLCN6 | 2.41 | 0.97 | 0.40 | 0.003 | down |
| TNRC6C | 4.02 | 1.93 | 0.48 | 0.003 | down |
| ZNRF3 | 4.29 | 1.81 | 0.42 | 0.003 | down |
| SLC25A11 | 14.28 | 6.26 | 0.44 | 0.003 | down |
| ATXN2 | 4.25 | 2.12 | 0.50 | 0.003 | down |
| KCNK6 | 2.23 | 0.72 | 0.32 | 0.003 | down |
| ERCC2 | 10.22 | 5.00 | 0.49 | 0.003 | down |
| RAB11FIP5;SFXN5 | 2.79 | 1.28 | 0.46 | 0.003 | down |
| FOXO3 | 6.99 | 2.57 | 0.37 | 0.003 | down |
| SIPA1L1 | 3.33 | 1.61 | 0.48 | 0.003 | down |
| MARC2 | 2.95 | 1.02 | 0.35 | 0.003 | down |
| AGAP1 | 2.14 | 0.94 | 0.44 | 0.003 | down |
| RPAP2 | 1.86 | 0.75 | 0.40 | 0.003 | down |
| SLC25A13 | 6.12 | 2.71 | 0.44 | 0.003 | down |
| HOMER1 | 4.21 | 1.87 | 0.44 | 0.003 | down |
| MAGI1 | 1.98 | 0.84 | 0.42 | 0.003 | down |
| ASPSCR1 | 2.42 | 1.09 | 0.45 | 0.003 | down |
| WDR70 | 2.99 | 1.31 | 0.44 | 0.003 | down |
| PARVA | 3.81 | 1.55 | 0.41 | 0.003 | down |
| EME2 | 3.06 | 1.28 | 0.42 | 0.003 | down |
| NDUFAF3 | 9.66 | 4.06 | 0.42 | 0.003 | down |
| GUSBP11 | 1.43 | 0.51 | 0.35 | 0.003 | down |
| TAF1 | 4.07 | 1.99 | 0.49 | 0.003 | down |
| TRIM35 | 14.67 | 6.65 | 0.45 | 0.003 | down |
| KDSR | 3.28 | 1.54 | 0.47 | 0.003 | down |
| BRD1 | 8.92 | 4.43 | 0.50 | 0.003 | down |
| CRIM1 | 13.59 | 6.66 | 0.49 | 0.003 | down |
| PHTF2 | 3.90 | 1.68 | 0.43 | 0.003 | down |
| AGAP2 | 2.89 | 1.21 | 0.42 | 0.004 | down |
| DBNL;AC017116.1 | 3.88 | 1.84 | 0.47 | 0.004 | down |
| MRPS34 | 38.33 | 11.33 | 0.30 | 0.004 | down |
| NFIB | 1.16 | 0.45 | 0.39 | 0.004 | down |
| NEK6 | 8.81 | 4.20 | 0.48 | 0.004 | down |
| RPUSD1 | 19.24 | 9.21 | 0.48 | 0.004 | down |
| SNPH | 1.48 | 0.53 | 0.36 | 0.004 | down |
| ARHGEF3 | 3.19 | 1.42 | 0.45 | 0.004 | down |
| NDUFAF8 | 18.60 | 7.06 | 0.38 | 0.004 | down |
| MAST3 | 3.46 | 1.54 | 0.45 | 0.004 | down |
| SSH2 | 1.81 | 0.77 | 0.43 | 0.004 | down |
| SULF2 | 3.18 | 1.42 | 0.45 | 0.004 | down |
| CDKL5 | 0.11 | 0.03 | 0.24 | 0.004 | down |
| POLR3H | 6.54 | 3.11 | 0.48 | 0.004 | down |
| KAZN | 1.62 | 0.72 | 0.45 | 0.004 | down |
| SLC2A13 | 0.53 | 0.17 | 0.32 | 0.004 | down |
| TCTA | 8.59 | 2.67 | 0.31 | 0.004 | down |
| ALDH1B1 | 10.08 | 1.41 | 0.14 | 0.005 | down |
| POLA1 | 7.26 | 3.59 | 0.49 | 0.005 | down |
| DGKZP1 | 4.39 | 1.65 | 0.38 | 0.005 | down |
| RAB11FIP3 | 6.64 | 3.15 | 0.47 | 0.005 | down |
| CMTM7 | 9.68 | 4.51 | 0.47 | 0.005 | down |
| LRP1 | 2.47 | 1.20 | 0.49 | 0.005 | down |
| PDE4A | 4.97 | 2.34 | 0.47 | 0.005 | down |
| TFAP4 | 3.67 | 1.72 | 0.47 | 0.005 | down |
| KIRREL1 | 3.90 | 1.88 | 0.48 | 0.005 | down |
| LAMC3 | 1.65 | 0.65 | 0.39 | 0.005 | down |
| CAMK1D | 2.62 | 1.21 | 0.46 | 0.005 | down |
| ATG4A | 3.47 | 1.32 | 0.38 | 0.005 | down |
| METTL26 | 11.45 | 5.02 | 0.44 | 0.005 | down |
| PC | 2.49 | 1.03 | 0.41 | 0.006 | down |
| PPARGC1B | 2.66 | 1.10 | 0.41 | 0.006 | down |
| AHCYL2 | 2.63 | 1.14 | 0.43 | 0.006 | down |
| SLC12A6 | 3.33 | 1.57 | 0.47 | 0.006 | down |
| CLMP | 1.93 | 0.76 | 0.40 | 0.006 | down |
| SHANK3 | 5.01 | 2.32 | 0.46 | 0.006 | down |
| MICALL2 | 2.11 | 0.94 | 0.44 | 0.006 | down |
| AMOTL1 | 4.77 | 1.83 | 0.38 | 0.006 | down |
| TMEM186 | 2.97 | 0.74 | 0.25 | 0.006 | down |
| FAAP100 | 7.46 | 3.50 | 0.47 | 0.006 | down |
| CCDC3 | 2.56 | 1.02 | 0.40 | 0.006 | down |
| TATDN2P2 | 1.59 | 0.50 | 0.31 | 0.006 | down |
| OGG1 | 1.24 | 0.50 | 0.40 | 0.006 | down |
| TIAM1 | 3.47 | 1.70 | 0.49 | 0.007 | down |
| AL669831.1 | 3.51 | 1.63 | 0.47 | 0.007 | down |
| RAPGEF2 | 1.85 | 0.87 | 0.47 | 0.007 | down |
| MIER2 | 3.53 | 1.69 | 0.48 | 0.007 | down |
| SCARA3 | 24.67 | 11.66 | 0.47 | 0.007 | down |
| ONECUT2 | 0.71 | 0.29 | 0.41 | 0.007 | down |
| ID1 | 86.81 | 39.75 | 0.46 | 0.007 | down |
| ARID1B | 1.07 | 0.54 | 0.50 | 0.007 | down |
| PCYOX1 | 4.99 | 1.98 | 0.40 | 0.007 | down |
| ARHGEF28 | 0.59 | 0.24 | 0.40 | 0.007 | down |
| ZNF767P | 1.38 | 0.47 | 0.34 | 0.007 | down |
| EIPR1 | 3.15 | 1.43 | 0.45 | 0.007 | down |
| GALK2 | 1.12 | 0.44 | 0.39 | 0.007 | down |
| CWC27 | 7.76 | 3.79 | 0.49 | 0.007 | down |
| TRAPPC12 | 2.24 | 1.04 | 0.46 | 0.008 | down |
| BTBD9 | 1.17 | 0.49 | 0.42 | 0.008 | down |
| SLC29A2 | 9.66 | 4.57 | 0.47 | 0.008 | down |
| SECTM1 | 5.24 | 2.34 | 0.45 | 0.008 | down |
| EEF1A1P6 | 87.19 | 4.46 | 0.05 | 0.008 | down |
| AP3M2 | 3.41 | 1.53 | 0.45 | 0.008 | down |
| HECTD2 | 1.12 | 0.48 | 0.43 | 0.008 | down |
| UST | 1.76 | 0.63 | 0.36 | 0.008 | down |
| PUSL1 | 10.41 | 4.78 | 0.46 | 0.008 | down |
| ANK1 | 0.34 | 0.11 | 0.32 | 0.008 | down |
| PANX1 | 7.39 | 2.95 | 0.40 | 0.009 | down |
| PLCG2 | 1.48 | 0.69 | 0.47 | 0.009 | down |
| GDF11 | 2.07 | 0.65 | 0.31 | 0.009 | down |
| BCL2L11 | 3.53 | 1.35 | 0.38 | 0.009 | down |
| LCMT1 | 6.25 | 2.84 | 0.46 | 0.009 | down |
| RNF7 | 5.22 | 1.71 | 0.33 | 0.009 | down |
| CSPG4 | 5.64 | 2.71 | 0.48 | 0.009 | down |
| THAP12 | 6.16 | 2.13 | 0.35 | 0.009 | down |
| COL27A1 | 1.26 | 0.58 | 0.46 | 0.009 | down |
| LIX1L | 8.03 | 3.98 | 0.50 | 0.009 | down |
| MAPKBP1 | 2.41 | 1.16 | 0.48 | 0.009 | down |
| NCKAP5L | 2.03 | 0.91 | 0.45 | 0.009 | down |
| SLC9A3R1;MIR3615 | 33.35 | 16.62 | 0.50 | 0.009 | down |
| CD99L2 | 1.54 | 0.62 | 0.40 | 0.009 | down |
| CRISPLD2 | 0.73 | 0.25 | 0.34 | 0.009 | down |
| GNG11 | 12.84 | 1.33 | 0.10 | 0.009 | down |
| LINC01001 | 1.84 | 0.72 | 0.39 | 0.010 | down |
| CHTF18 | 5.91 | 2.92 | 0.49 | 0.010 | down |
| APOL2 | 4.86 | 2.27 | 0.47 | 0.010 | down |
| MAP3K20 | 5.54 | 2.41 | 0.43 | 0.010 | down |
| AC121761.1 | 2.71 | 0.92 | 0.34 | 0.011 | down |
| OPLAH | 4.63 | 2.10 | 0.45 | 0.011 | down |
| PI4KAP2 | 1.77 | 0.78 | 0.44 | 0.011 | down |
| KIAA1024 | 1.22 | 0.48 | 0.39 | 0.011 | down |
| BPHL | 1.69 | 0.72 | 0.42 | 0.011 | down |
| SLC39A4 | 5.35 | 2.64 | 0.49 | 0.011 | down |
| PTPRN2 | 1.19 | 0.44 | 0.37 | 0.011 | down |
| DNAJC22 | 5.13 | 2.55 | 0.50 | 0.011 | down |
| MROH1 | 3.05 | 1.51 | 0.49 | 0.011 | down |
| UVSSA | 2.13 | 0.88 | 0.41 | 0.011 | down |
| INPP5A | 4.21 | 1.96 | 0.47 | 0.011 | down |
| RABL2B | 3.93 | 1.86 | 0.47 | 0.011 | down |
| B3GLCT | 1.89 | 0.80 | 0.42 | 0.011 | down |
| BRK1 | 39.06 | 17.88 | 0.46 | 0.011 | down |
| KAT6B | 1.92 | 0.93 | 0.49 | 0.011 | down |
| ABHD17C | 6.76 | 3.36 | 0.50 | 0.012 | down |
| MAFG-AS1 | 3.05 | 0.84 | 0.27 | 0.012 | down |
| COBL | 1.03 | 0.48 | 0.46 | 0.012 | down |
| ABHD11 | 8.15 | 3.91 | 0.48 | 0.012 | down |
| PITHD1 | 23.37 | 11.38 | 0.49 | 0.012 | down |
| DOPEY2 | 1.80 | 0.84 | 0.47 | 0.012 | down |
| TGFB2 | 0.33 | 0.09 | 0.28 | 0.012 | down |
| UCP2 | 4.29 | 1.92 | 0.45 | 0.012 | down |
| ADARB1 | 2.10 | 1.02 | 0.48 | 0.012 | down |
| GINS4 | 2.89 | 1.34 | 0.46 | 0.013 | down |
| AC009093.9 | 0.88 | 0.19 | 0.21 | 0.013 | down |
| NHSL1 | 1.44 | 0.66 | 0.46 | 0.013 | down |
| PRICKLE3 | 5.30 | 2.40 | 0.45 | 0.013 | down |
| LIMS2 | 0.55 | 0.20 | 0.37 | 0.013 | down |
| PROM1 | 2.02 | 0.93 | 0.46 | 0.013 | down |
| NECAB3 | 4.11 | 1.87 | 0.46 | 0.013 | down |
| FBXO41 | 7.01 | 3.23 | 0.46 | 0.013 | down |
| LRRC3 | 1.17 | 0.27 | 0.23 | 0.013 | down |
| SCAND1 | 6.70 | 2.30 | 0.34 | 0.013 | down |
| SLC9A3R2 | 7.34 | 3.58 | 0.49 | 0.013 | down |
| SLC2A6 | 5.02 | 2.32 | 0.46 | 0.013 | down |
| GALK1 | 6.79 | 3.26 | 0.48 | 0.013 | down |
| FOXRED1 | 4.44 | 2.18 | 0.49 | 0.014 | down |
| AL359075.1 | 0.96 | 0.36 | 0.37 | 0.014 | down |
| LRRC20 | 2.91 | 1.13 | 0.39 | 0.014 | down |
| ETV6 | 1.83 | 0.76 | 0.42 | 0.014 | down |
| COMMD8 | 9.35 | 4.38 | 0.47 | 0.015 | down |
| C10orf76 | 5.52 | 2.66 | 0.48 | 0.015 | down |
| RNF121 | 2.75 | 1.18 | 0.43 | 0.015 | down |
| NCK2 | 6.20 | 2.86 | 0.46 | 0.015 | down |
| YKT6 | 32.83 | 16.24 | 0.49 | 0.015 | down |
| RLIM | 3.99 | 1.10 | 0.28 | 0.016 | down |
| FAM20B | 10.87 | 4.60 | 0.42 | 0.016 | down |
| BCAS3 | 0.56 | 0.24 | 0.43 | 0.016 | down |
| SYT12 | 0.90 | 0.38 | 0.43 | 0.016 | down |
| MCUB | 4.48 | 2.14 | 0.48 | 0.016 | down |
| RASSF3 | 20.59 | 7.83 | 0.38 | 0.017 | down |
| ARPC5L | 9.34 | 4.54 | 0.49 | 0.017 | down |
| TRIM4 | 3.02 | 1.28 | 0.43 | 0.017 | down |
| PGM2L1 | 1.04 | 0.45 | 0.43 | 0.017 | down |
| TPPP | 1.96 | 0.52 | 0.26 | 0.017 | down |
| PIP5KL1 | 0.96 | 0.32 | 0.33 | 0.017 | down |
| TGFB1I1 | 2.69 | 1.28 | 0.48 | 0.017 | down |
| BIK | 9.06 | 3.89 | 0.43 | 0.018 | down |
| AL162586.1 | 0.51 | 0.15 | 0.29 | 0.018 | down |
| RNASEH2B | 2.01 | 1.00 | 0.50 | 0.018 | down |
| WNT10B | 4.55 | 1.67 | 0.37 | 0.018 | down |
| PAXX | 16.44 | 8.02 | 0.49 | 0.018 | down |
| RALGAPA2 | 0.61 | 0.25 | 0.41 | 0.018 | down |
| MT-RNR2 | 10.66 | 5.08 | 0.48 | 0.018 | down |
| DMAC1 | 3.09 | 1.39 | 0.45 | 0.018 | down |
| RALGPS1 | 0.42 | 0.16 | 0.38 | 0.018 | down |
| TCF19 | 10.07 | 4.64 | 0.46 | 0.019 | down |
| RHPN1 | 2.53 | 1.10 | 0.44 | 0.019 | down |
| RGS19 | 8.63 | 4.16 | 0.48 | 0.019 | down |
| SLC25A17 | 3.69 | 1.76 | 0.48 | 0.019 | down |
| HNRNPA1P10 | 33.01 | 10.17 | 0.31 | 0.019 | down |
| NFE2L3 | 5.03 | 2.02 | 0.40 | 0.019 | down |
| FRY | 0.07 | 0.02 | 0.28 | 0.019 | down |
| NPW | 6.61 | 1.73 | 0.26 | 0.020 | down |
| TMEM53 | 0.92 | 0.25 | 0.27 | 0.020 | down |
| STXBP4 | 1.50 | 0.70 | 0.47 | 0.021 | down |
| ATP2A3 | 2.16 | 1.04 | 0.48 | 0.021 | down |
| DNAH3 | 0.05 | 0.01 | 0.27 | 0.021 | down |
| MARCH9 | 1.20 | 0.47 | 0.39 | 0.021 | down |
| ORMDL2 | 5.57 | 2.71 | 0.49 | 0.022 | down |
| NF1 | 0.72 | 0.36 | 0.50 | 0.022 | down |
| ZNF91 | 0.28 | 0.08 | 0.27 | 0.022 | down |
| ATP8B3 | 2.31 | 1.15 | 0.50 | 0.022 | down |
| TEC | 1.48 | 0.62 | 0.42 | 0.022 | down |
| AC240565.1 | 0.57 | 0.16 | 0.29 | 0.023 | down |
| AGAP5;BMS1P4;AC022400.3;AC022400.5 | 0.88 | 0.36 | 0.41 | 0.023 | down |
| JMJD4 | 2.05 | 0.88 | 0.43 | 0.023 | down |
| CGNL1 | 0.64 | 0.26 | 0.41 | 0.023 | down |
| RBMS2P1 | 1.36 | 0.14 | 0.10 | 0.023 | down |
| UBE2R2 | 14.11 | 5.14 | 0.36 | 0.024 | down |
| ZNF283 | 1.13 | 0.44 | 0.39 | 0.025 | down |
| ARMC4 | 0.90 | 0.37 | 0.41 | 0.025 | down |
| C2orf54 | 2.36 | 0.95 | 0.40 | 0.025 | down |
| SCHIP1 | 0.16 | 0.03 | 0.18 | 0.025 | down |
| CLDN4 | 9.33 | 4.36 | 0.47 | 0.025 | down |
| DBNDD1 | 13.12 | 5.48 | 0.42 | 0.025 | down |
| LINC01002 | 0.49 | 0.19 | 0.40 | 0.025 | down |
| MAGEB2 | 38.78 | 8.08 | 0.21 | 0.026 | down |
| OSBPL5 | 1.54 | 0.73 | 0.48 | 0.026 | down |
| CDRT4 | 1.09 | 0.26 | 0.24 | 0.026 | down |
| MICALCL | 0.42 | 0.14 | 0.32 | 0.026 | down |
| LCOR | 4.90 | 2.33 | 0.47 | 0.026 | down |
| PTCH2 | 0.76 | 0.31 | 0.41 | 0.026 | down |
| SS18L2 | 3.44 | 1.17 | 0.34 | 0.026 | down |
| SLC12A8 | 2.55 | 1.24 | 0.48 | 0.026 | down |
| CCDC189 | 0.79 | 0.28 | 0.35 | 0.027 | down |
| TRAPPC6A | 2.60 | 0.90 | 0.35 | 0.027 | down |
| TSPAN9 | 2.33 | 1.12 | 0.48 | 0.027 | down |
| TLCD1 | 6.79 | 3.20 | 0.47 | 0.028 | down |
| CCND3 | 7.38 | 3.60 | 0.49 | 0.028 | down |
| FAM157B | 0.52 | 0.17 | 0.32 | 0.028 | down |
| FAM129A | 1.32 | 0.64 | 0.48 | 0.028 | down |
| PHLPP1 | 1.58 | 0.78 | 0.49 | 0.029 | down |
| FAM172A;POU5F2 | 0.63 | 0.27 | 0.43 | 0.029 | down |
| SLC10A7 | 0.61 | 0.26 | 0.42 | 0.030 | down |
| ATP7B | 0.93 | 0.43 | 0.47 | 0.030 | down |
| PCDH7 | 6.64 | 3.28 | 0.49 | 0.030 | down |
| PPM1M | 2.20 | 1.00 | 0.45 | 0.030 | down |
| MT-ND2 | 10.57 | 5.05 | 0.48 | 0.030 | down |
| NMNAT1;AL603962.1 | 2.40 | 1.19 | 0.49 | 0.031 | down |
| PNMA2 | 0.32 | 0.12 | 0.36 | 0.031 | down |
| PCSK6 | 0.59 | 0.27 | 0.47 | 0.032 | down |
| RUBCNL | 0.30 | 0.11 | 0.36 | 0.032 | down |
| BRICD5 | 1.50 | 0.55 | 0.37 | 0.032 | down |
| CABLES2 | 4.60 | 2.25 | 0.49 | 0.032 | down |
| KSR1 | 0.60 | 0.27 | 0.45 | 0.032 | down |
| LHFPL2 | 2.16 | 1.02 | 0.47 | 0.033 | down |
| ZNF530 | 0.59 | 0.18 | 0.30 | 0.033 | down |
| TENM3 | 0.89 | 0.44 | 0.50 | 0.034 | down |
| SPIRE2 | 1.43 | 0.71 | 0.50 | 0.034 | down |
| FAM157C | 0.56 | 0.21 | 0.38 | 0.034 | down |
| ZNF324B | 0.74 | 0.26 | 0.35 | 0.035 | down |
| NETO2 | 2.81 | 1.34 | 0.48 | 0.035 | down |
| CACNA1I | 0.07 | 0.02 | 0.29 | 0.035 | down |
| LDLRAD3 | 5.69 | 2.20 | 0.39 | 0.035 | down |
| TBX1 | 0.24 | 0.07 | 0.30 | 0.035 | down |
| ZNF543 | 0.98 | 0.25 | 0.26 | 0.036 | down |
| ANKRD9 | 0.74 | 0.30 | 0.40 | 0.037 | down |
| C16orf74 | 3.80 | 1.75 | 0.46 | 0.037 | down |
| TBXA2R | 1.53 | 0.61 | 0.40 | 0.038 | down |
| LINC00640 | 0.64 | 0.21 | 0.32 | 0.038 | down |
| STARD13 | 0.69 | 0.30 | 0.44 | 0.038 | down |
| ZNF594 | 0.34 | 0.13 | 0.37 | 0.039 | down |
| ZNF460 | 0.55 | 0.18 | 0.32 | 0.039 | down |
| RNF168 | 7.16 | 3.31 | 0.46 | 0.039 | down |
| ZDHHC14 | 0.44 | 0.19 | 0.43 | 0.039 | down |
| GRIN1 | 1.03 | 0.49 | 0.47 | 0.039 | down |
| PYCR3 | 2.96 | 1.27 | 0.43 | 0.040 | down |
| TMEM121 | 2.69 | 1.17 | 0.43 | 0.040 | down |
| RBM20 | 0.48 | 0.20 | 0.43 | 0.040 | down |
| UBE2E2 | 1.93 | 0.90 | 0.47 | 0.041 | down |
| CAV1 | 15.10 | 6.79 | 0.45 | 0.041 | down |
| GNB1L | 0.84 | 0.38 | 0.45 | 0.041 | down |
| PPM1H | 3.32 | 1.60 | 0.48 | 0.041 | down |
| SLC35E2B | 4.24 | 1.75 | 0.41 | 0.042 | down |
| AC007038.1 | 0.43 | 0.13 | 0.29 | 0.042 | down |
| SLC35C1 | 6.57 | 3.11 | 0.47 | 0.042 | down |
| CCDC86 | 19.72 | 9.34 | 0.47 | 0.042 | down |
| C1GALT1C1 | 2.95 | 0.79 | 0.27 | 0.043 | down |
| CHN1 | 0.83 | 0.38 | 0.45 | 0.043 | down |
| TMEM143 | 2.14 | 1.00 | 0.47 | 0.043 | down |
| MRGPRF | 0.57 | 0.21 | 0.36 | 0.043 | down |
| NEBL | 0.40 | 0.17 | 0.43 | 0.045 | down |
| AC108010.1 | 2.87 | 1.25 | 0.44 | 0.045 | down |
| CAVIN3 | 7.11 | 2.79 | 0.39 | 0.046 | down |
| EMC3-AS1 | 1.13 | 0.37 | 0.33 | 0.047 | down |
| ZNF628 | 0.84 | 0.20 | 0.24 | 0.047 | down |
| DGAT2 | 0.30 | 0.12 | 0.38 | 0.047 | down |
| EVL | 0.55 | 0.26 | 0.47 | 0.048 | down |
| MARVELD3 | 1.71 | 0.70 | 0.41 | 0.049 | down |
| CLIC3 | 3.89 | 1.69 | 0.44 | 0.049 | down |
| TNFRSF11A | 0.50 | 0.23 | 0.45 | 0.049 | down |
| ABCC6 | 0.39 | 0.15 | 0.39 | 0.050 | down |
